# Genome size variation and polyploidy prevalence in the genus *Eragrostis* are associated with the global dispersal in arid area

**DOI:** 10.3389/fpls.2023.1066925

**Published:** 2023-03-13

**Authors:** Ge-Ran Hutang, Yan Tong, Xun-Ge Zhu, Li-Zhi Gao

**Affiliations:** ^1^ Germplasm Bank of Wild Species in Southwestern China, Kunming Institute of Botany, Chinese Academy of Sciences, Kunming, Yunnan, China; ^2^ University of Chinese Academy of Sciences, Beijing, China; ^3^ Engineering Research Center for Selecting and Breeding New Tropical Crop Varieties, Ministry of Education, College of Tropical Crops, Hainan University, Haikou, China

**Keywords:** genome size, phylogeny, *Eragrostis*, adaptation, environmental factors

## Abstract

**Background:**

Biologists have long debated the drivers of the genome size evolution and variation ever since Darwin. Assumptions for the adaptive or maladaptive consequences of the associations between genome sizes and environmental factors have been proposed, but the significance of these hypotheses remains controversial. *Eragrostis* is a large genus in the grass family and is often used as crop or forage during the dry seasons. The wide range and complex ploidy levels make *Eragrostis* an excellent model for investigating how the genome size variation and evolution is associated with environmental factors and how these changes can ben interpreted.

**Methods:**

We reconstructed the *Eragrostis* phylogeny and estimated genome sizes through flow cytometric analyses. Phylogenetic comparative analyses were performed to explore how genome size variation and evolution is related to their climatic niches and geographical ranges. The genome size evolution and environmental factors were examined using different models to study the phylogenetic signal, mode and tempo throughout evolutionary history.

**Results:**

Our results support the monophyly of *Eragrostis*. The genome sizes in *Eragrostis* ranged from ~0.66 pg to ~3.80 pg. We found that a moderate phylogenetic conservatism existed in terms of the genome sizes but was absent from environmental factors. In addition, phylogeny-based associations revealed close correlations between genome sizes and precipitation-related variables, indicating that the genome size variation mainly caused by polyploidization may have evolved as an adaptation to various environments in the genus *Eragrostis*.

**Conclusion:**

This is the first study to take a global perspective on the genome size variation and evolution in the genus *Eragrostis*. Our results suggest that the adaptation and conservatism are manifested in the genome size variation, allowing the arid species of *Eragrostis* to spread the xeric area throughout the world.

## Introduction

Genome size is a fundamental biological characteristic for the organism. Variation in genome size was thought to have “functional consequences” correlated with the environmental conditions and individual phenotypes (Knight et al., 2005; Moraes et al., 2022). Thus, the genome size variation is an essential parameter to understand the evolutionary models of the species. Genome sizes are also affected by environmental factors as well (Faizullah et al., 2021). The nuclear genome size varied among land plants with 12-fold in mosses to 2,342-fold in angiosperms (Leitch and Leitch, 2013), evolutionary and environmental implications behind this diversity are still largely unknown. Up to now, opinions on increasing or decreasing genome size could be divided into two types: (1) plants with small genomes adapted to all environmental conditions, while those with large genomes were limited in a narrow area and tended to be excluded from extreme habitats for the short growing season (Knight and Ackerly, 2002) and redundant noncoding DNA (Vinogradov, 2003; Knight et al., 2005; Pyšek et al., 2018); and (2) species with large genomes were also found in more seasonal or arid areas as they have chosen a mechanism of cell expansion rather than rapid division to cope with extreme environments (Grime and Mowforth, 1982). As Bennett (1987) indicated, large genomes have enabled plants to generate superior traits in adverse habitats. In addition, evolution is an efficient way to elucidate genome size variation with the “adaptive theory” or “junk DNA” theory (Petrov, 2001; Pellicer et al., 2018). To tackle the questions raised above, phylogenetic comparative methods (PCMs) are increasingly used in evolutionary biology to test evolutionary associations between genome sizes and environmental conditions and resolve the confusion over the consequences of additional DNA in genome size. Moreover, the evolutionary process of bioclimatic niches and geographical factors may reflect conservation, diversification, and adaptation in a phylogenetic context (Butler and King, 2004; Kozak and Wiens, 2010; Wiens et al., 2010). For example, studies on “phylogenetic niche conservatism” (PNC) (Wiens and Graham, 2005; Losos, 2008) have paid more attention to the variation of environmental factors. It is a potential explanation of the species adapted to new or changing environments (Ackerly, 2003). However, the ecological factors that determine evolutionary models leading to the genome size variation are yet to be fully elucidated.

*Eragrostis* Wolf, the largest genus comprising more than 350 species in the subfamily Chloridoideae (Poaceae) (Van den Borre and Watson, 1994), is widely distributed in diverse ecological environments. *Eragrostis* species often grow on sandy, clay, rocky slopes and gravel soils. The majority of *Eragrostis* species are common grasses without clear use, and only a few species are used as forage grasses. *Eragrostis* species can maintain high productivity even under arid or intensive grazing conditions, leading to some species deemed as “miracle grass” in the last century (Cox et al., 1988). Some *Eragrostis* species have been used for soil conservation and fodder crops in Africa, America and Australia (Zeid et al., 2011). In Africa, wild grains of some species were used as famine food by humans (Watt and Breyer-Brandwijk, 1962; Burkill, 1995). Cereal crops that can grow under various stresses with minimal agricultural inputs are essential (Assefa et al., 2017). *Eragrostis tef* (Zucc.) Trotter, commonly known as Teff, originated in Ethiopia, and fills this pivotal role due to nutritional merits and tolerance to harsh environments, becoming a healthy alternative food in the xeric areas (Alaunyte et al., 2012; Abraham, 2015; Cheng, 2018; McSteen and Kellogg, 2022). Due to the global climate changes, the desertification zone is expanding slowly, making it urgent to mine the untapped potential of plant species in drylands (Brown, 2012). Thus, this genus is well suited to study drought adaptation for its remarkable viability in dry habitats (Colom and Vazzana, 2001; Balsamo et al., 2006; Degu et al., 2008; Ginbot and Farrant, 2011; Buerdsell et al., 2022). Additionally, *Eragrostis* species have varied ploidy levels, which are recognized as a major driver of genomic variation. However, there are only three species in genus *Eragrostis* have been measured in previous studies. (http://data.kew.org/cvalues). Thus, the genus *Eragrostis* is a good model to test the genome size variation and evolution in a global context and to find the correlations between genome sizes and environmental factors.

The monophyletic nature of the genus *Eragrostis* has been controversial for years (Van den Borre and Watson, 1994; Ingram and Doyle, 2007; Ingram, 2010; Girma et al., 2018). Ingram and Doyle (2007) attributed this confusion to the large number of species in *Eragrostis* and its unclear phylogenetic relationship with closely related genera. However, some classifications in *Eragrostis* have been proposed. The genus was divided into several subgroups with various anatomical types (Ellis, 1977; Prendergast et al., 1986; Van den Borre and Watson, 1994; Ingram, 2010). Molecular phylogenetic analyses were recently performed based on plastid and nuclear data using limited samples of *Eragrostis* (Ingram and Doyle, 2004; Ingram and Doyle, 2007; Somaratne et al., 2019; Liu et al., 2021). Although Barrett et al. (2020) investigated 118 species of *Eragrostidinae*, they primarily put emphasis on the phylogeny of *Eragrostis* in Australia. Another contentious question in the phylogenetic relationship of *Eragrostis* is the presumed progenitors of *E. tef* (allotetraploid; 2n = 4x = 40). Although it has been long recognized that *E. tef* was originated from two diploid progenitors, the true progenitors of *E. tef* were still ambiguous (Jones et al., 1978; Doyle, 1999; Ingram and Doyle, 2003). Up to eighteen species have been reported as the presumed ancestors of *E. tef* (Girma et al., 2018). Among these presumed ancestors, *E. pilosa* has a close relationship with *E. tef* (Costanza et al., 1979; Ketema, 1991; Ayele et al., 1999; Ayele and Nguyen, 2000; Tefera et al., 2001; Ingram and Doyle, 2003). However, Girma et al. (2018) proposed that *E. pilosa* could be an intermediate progenitor, providing robust evidence for *E. aethiopica*, a diploid species (2n = 2x =20) (Bekele and Lester, 1981; Tavassoli, 1986), being the progenitor close to *E. tef* and *E. pilosa*. A well-resolved phylogenetic tree of this genus will greatly help to determine the diploid ancestor of *E. tef*.

In this study, we reconstructed the phylogeny of *Eragrostis* using plastid data and ITS regions based on 66 representative samples from the worldwide distributions. ITS region is often used to trace the origin and evolution of allopolyploids at low taxonomic level (Soltis et al., 2008), and plastid data can also be employed to determine the maternal parents of *E. tef* assuming their maternally inherited attribution in grasses (Mogensen, 1988). We further estimated genome sizes of *Eragrostis* 66 samples based on flow cytometric experiments, including previously identified taxa (*E. spectabilis*, *E. minor* and *E. tef*) for comparisons. We investigated genome size variation across clades, extending to the distributions, ranges and life styles. We tried to demonstrate the evolutionary models of genome sizes and environmental factors (such as geographical distributions and climatic niches) based on tempo, mode and phylogenetic signal in evolution in an attempt to select the best model through history. We evaluated the correlations between genome sizes and environmental factors in a phylogenetic context, which is capable of revealing environmental forces in shaping genome size variation. Finally, we reconstructed the ancestral states of climatic niche correlates to see environmental changes of *Eragrostis* species. Throughout the study, we reviewed the phylogeny and environmental conditions of each *Eragrostis* species, given the implications for the response of *Eragrostis* species to future climate changes.

## Materials and Methods

### Taxon sampling and DNA sequencing

Most *Eragrostis* species are native to Africa, Australia and America (Cufodontis, 1953; Costanza, 1978; Lazarides, 1997). Our samples not only covered the above-mentioned distribution, but also included those species from Europe and Asia. We summarized the information of 66 individuals representing 47 species (*E. cylindriflora* with 3 samples; *E. tef* with 18 cultivars) in this study (see **Tables 1**, **S1**). Seeds were germinated in pots filled with sterile soil, and placed in a naturally lit glasshouse (Kunming Institute of Botany, Chinese Academy of Science). In order to evaluate the data accuracy, we planted the samples in 2017 and 2020, respectively. Total genomic DNA was isolated from fresh leaves following a modified cetyltrimethylammonium bromide (CTAB) protocol (Li et al., 2013). One nuclear locus (ITS) and three chloroplast regions (*rbcL*, *matK*, *trnL-trnF*) were employed for DNA sequencing. The primers used for polymerase chain reactions (PCR) and sequencing are presented in **Table S2**. The PCR experiments were performed in a 27 μl volume containing 13.5 μl of 2 × Taq polymerase mix (Tiangen Biotech), 1.5 μl of each primer, 1.5 μl of DNA template and 9 μl sterile deionized water. The amplification parameters for all regions according to the following protocol: 94°C for 5 min, followed by 35 cycles of 94°C for 1min, 45°C for 1min, 72°C for 1 min 30 s, and a final 10 min extension at 72°C. The PCR products were cleaned using polyethylene glycol (PEG) precipitation protocol (Wen et al., 2007). DNA sequencing was performed with BigDye Terminator Cycle Sequencing kit (Applied Biosystems, Foster City, CA, USA) and ABI PRISM 3730 genetic analyzer (ABI). The program Sequencer 4.8 (Gene Code Corporation) was employed to assemble the generated sequences.

**Table 1:** Summary of *Eragrostis* species included in this study.

Species	2C (pg)	SD (pg)	1C (Mbp)	Estimated Ploidy level	Annual Precipitation (mm)	Chromosome numbers^a^ 2n/DNA content (2C, pg)
*Eragrostis acutiflora*	2.99	0.56	1463.32	2n=10x=100	2359	40^1,2^
*Eragrostis acutiglumis*	2.78	0.40	1358.14	2n=8x=80	1281	
*Eragrostis aethiopica* *	0.69	0.11	335.46	2n=2x=20	608	20^50^
*Eragrostis bahiensis*	2.32	0.25	1134.75	2n=8x=80	1404	
*Eragrostis barbinodis*	2.06	0.14	1007.29	2n=6x=60	624	40^4,5,6^, 50^3^
*Eragrostis bicolor* *	1.98	0.10	968.87	2n=6x=60	356	
*Eragrostis cilianensis* *	0.77	0.03	376.69	2n=2x=20	670	20^7,8,9,50^, 40^10^
*Eragrostis cylindriflora*	1.93	0.16	941.88	2n=6x=60	486	
*Eragrostis dielsii*	2.58	0.14	1263.08	2n=8x=80	259	
*Eragrostis echinochloidea*	1.46	0.04	714.37	2n=4x=40	377	40^4,5,12,13^, 60^11^
*Eragrostis eriopoda*	3.80	0.06	1856.36	2n=12x=120	298	
*Eragrostis ferruginea* *	3.32	0.05	1621.19	2n=10x=100	1362	80^14^
*Eragrostis gummiflua*	2.94	0.22	1439.54	2n=10x=100	662	40^15,16^
*Eragrostis heteromera* *	1.69	0.11	826.20	2n=6x=60	720	40^4,5,6,12,15,16,^
*Eragrostis humidicola*	1.70	0.03	829.97	2n=6x=60	1268	
*Eragrostis intermedia*	3.55	0.10	1735.29	2n=12x=120	585	80^17,18^, c.120^17,18^
*Eragrostis japonica*	2.03	0.05	991.70	2n=6x=60	1075	20^19,20,^ 40^21,22^,60^23,24^
*Eragrostis lappula*	1.94	0.19	948.71	2n=6x=60	670	40^6,15,16^
*Eragrostis lehmanniana* *	1.91	0.07	932.07	2n=6x=60	417	40^3,7^, 60^3^
*Eragrostis leptocarpa*	1.88	0.06	918.11	2n=6x=60	296	
*Eragrostis lugens* *	2.37	0.04	1157.38	2n=8x=80	944	40^6^, 80^6^
*Eragrostis virescens*	1.98	0.15	969.88	2n=6x=60	705	
*Eragrostis minor* *	1.38	0.08	674.93	2n=4x=40	780	20^45^, 40^25,26^,80^27,28^ **/**1.46^46^
*Eragrostis neesii*	0.66	0.05	320.69	2n=2x=20	1438	
*Eragrostis nigra*	1.80	0.09	878.29	2n=6x=60	887	60^20,24,29^
*Eragrostis nindensis*	2.16	0.23	1054.11	2n=6x=60	441	
*Eragrostis nutans*	2.00	0.01	979.92	2n=6x=60	1677	40^10,22^, 60^23,30,31^
*Eragrostis obtusa* *	1.53	0.15	747.31	2n=4x=40	440	20^4,5,12^
*Eragrostis papposa* *	1.41	0.01	691.27	2n=4x=40	377	20^32,33^
*Eragrostis patens*	0.69	0.03	337.23	2n=2x=20	944	20^34,50^
*Eragrostis patentipilosa*	2.42	0.28	1185.4	2n=8x=80	716	
*Eragrostis patentissima*	2.06	0.04	1008.63	2n=6x=60	862	
*Eragrostis pilosa* *	1.97	0.11	962.67	2n=6x=60	842	20^35^, 40^27,36,37,38,39^
*Eragrostis plana*	2.10	0.15	1028.19	2n=6x=60	800	20^3^
*Eragrostis polytricha*	2.85	0.06	1395.88	2n=8x=80	1484	c.60^40^
*Eragrostis porosa*	1.35	0.05	658.69	2n=4x=40	325	
*Eragrostis racemosa*	1.79	0.09	877.73	2n=6x=60	838	40^4,5,12,19^, 60^6^
*Eragrostis rotifer*	1.42	0.06	696.35	2n=4x=40	444	40^41^
*Eragrostis rufescens*	1.46	0.08	715.05	2n=4x=40	1392	
*Eragrostis sarmentosa*	1.43	0.03	701.51	2n=4x=40	564	40^42^
*Eragrostis spectabilis*	2.17	0.44	1060.92	2n=6x=60	1050	20^43^, 40^13^ **/**2.30^47^
*Eragrostis superba*	1.59	0.08	775.29	2n=4x=40	622	20^3^, 40^3,4,5,12,15,24,29,37^
*Eragrostis tenella*	0.71	0.04	346.31	2n=2x=20	1456	20^50^
*Eragrostis tenuifolia*	1.35	0.01	660.14	2n=4x=40	1338	
*Eragrostis tremula*	2.11	0.09	1032.29	2n=6x=60	352	20^6,19,23^, 40^22,24^
*Eragrostis unioloides*	1.91	0.01	936.24	2n=6x=60	1892	20^20,23,30,29^, 60^10,22,24^
*Eragrostis tef*	1.28	0.093	624.00	2n=4x=40	787	40^4,5.12,13,24,44^ **/**1.40^44^, 1.28^48^,1.58^49^

The genome sizes of 18 cultivars in Eragrostis tef and other 2 samples in Eragrstis cylindriflora are displayed in **Table S7**. Arid species: The values of annual precipitation less than 800 mm were marked in red. *, asterisks represent presumed ancestors of Eragrostis tef in Girma et al. (2018). ^a^ chromosome numbers from references:^1^(Gould and Soderstrom, 1970a); ^2^(Davidse and Pohl, 1974); ^3^(Spies and Jonker, 1987); ^4^(Roodt and Spies, 2002); ^5^(Roodt and Spies, 2003a); ^6^(Cave, 1959a); ^7^(Femandes and Queiros, 1969); ^8^(Faruqi et al., 1987); ^9^(Bir, 1983); ^10^(Gohil, 1986); ^11^(Spies and Voges, 1988); ^12^(Roodt and Spies, 2003b); ^13^(Darlington and Wylie, 1956); ^14^(Tateoka and Löve, 1967); ^15^(JMJ, 1954); ^16^(Cave, 1959b); ^17^(Gould, 1968); ^18^(Reeder, 1971); ^19^(Ornduff, 1969); ^20^(Mehra, 1982); ^21^(Sahni and Bir, 1985); ^22^(Bir and Sahni, 1988); ^23^(Christopher and Abraham, 1974); ^24^(Kumar, 1987); ^25^(Majovsky, 1974); ^26^(Ghukasyan, 2004); ^27^(Devesa, 1990); ^28^(Moore, 1982); ^29^(Goldblatt, 1981); ^30^(Gould and Soderstrom, 1974); ^31^(Cave, 1962); ^32^(Löve, 1970); ^33^(Gould and Soderstrom, 1970b); ^34^(Dujardin and Tilquin, 1971); ^35^(Murin et al., 2000); ^36^(Marhold, 2006); ^37^(Nirmala and Rao, 1981); ^38^(Fernandes, 1971); ^39^(Chepinoga et al., 2009); ^40^(Gould and Soderstrom, 1967); ^41^(Nordenstam, 1969); ^42^(Spies et al., 1991); ^43^(Sherif et al., 1983); ^44^(Bennett and Smith, 1976); ^45^(Moinuddin et al., 1994); ^46^(Šmarda et al., 2014); ^47^(Bai et al., 2012); ^48^(VanBuren et al., 2020); ^49^(Cannarozzi et al., 2014); ^50^(Tavassoli, 1986).

### Phylogenetic analyses

A total of 66 individuals of *Eragrostis* and the four outgroup species were included in our phylogenetic analyses. Based on the results of Peterson et al. (2010), two species from Cotteinae (*Cottea pappophoroides*, *Enneapogon desvauxii*) and two species from Uniolinae (*Tetrachne dregei*, *Uniola paniculata*) were downloaded from NCBI to act as outgroups (**Table S3**). Sequences of ITS, *matK*, *trnL-trnF* and *rbcL* regions were aligned and combined in MUSCLE software (version 3.8.31) (Edgar, 2004). All sequences have been deposited in National Genomics Data Center under BioProject number CRA007592. We used maximum likelihood (ML) and Bayesian analyses to infer phylogenetic relationships for both separated and combined data. All gaps were treated as missing data. The ML analyses being conducted using RAxML software with GTR + I + G4 model selected in Modeltest-ng (Darriba et al., 2020). The bootstrap values (BS) were estimated with 1,000 replicates for internal branch support. The BS values of 90-100 were a statistically significant occurrence, while 89-90 was interpreted as a moderate support (Peterson et al., 2010). Phylogenetic analyses were also performed using the Bayesian analyses in MrBayes version 3.2.7 on the XSEDE CIPRES platform (Ronquist et al., 2012). The best model for the Bayesian analyses was selected in jModeltest 2.1.4 (Darriba et al., 2012) of TIM + I + G with a gamma shape (G) of 0.704. Each Bayesian analysis was run for 10,000,000 generations. The analysis was run until the value of the standard deviation of split frequencies dropped below 0.01. The percentage of the sampled values discarded as burn-in was set at 25%, and then the remaining trees were used to construct the consensus tree. The posterior probabilities (PP) of 0.95-1.00 were considered as significant probabilities (Peterson et al., 2010). The plastid and ITS data sets were separately processed in DnaSP version 6.12.03 software (Rozas et al., 2017) for calculating the characteristics of sequences. The final consensus trees were uploaded to iTOL (http://itol.embl.de) for editing and visualization.

### Genome size estimation

The prevalence of polyploids in the genus *Eragrostis* even exists within one species (Burson and Voigt, 1996), making it complicated to calculate the 1C DNA values, and thus, we interpreted 2C DNA content as genome size in this study. We measured genome sizes of all samples using two reference standards: *Oryza sativa* L. ssp. *japonica* cv. Nipponbare (389 Mbp) (Sasaki, 2005) and *Solanum lycopersicum* (900 Mbp) (Tomato Genome Consortium, 2012). The coefficient of variation (CV) less than 5% was considered as a reliable estimate. Nuclei suspensions were improved according to two-step procedure using Otto I and Otto II buffers (Otto, 1990). 5 cm^2^ pieces of young leaves of the samples and the standards were chopped separately, using a scalpel in a petri dish containing 1ml of Otto I buffer (1 M citric acid and 0.5% Tween 20) on ice. Samples were incubated at room temperature for 60 min and shaken lightly at the same time. Suspensions were filtered through a 50-μm nylon filter and nuclei were stained with 1 mL Otto II buffer (0.4 M Na_2_HPO_4_.12 H_2_0) supplemented with PI (final concentration 50 ug/ml) and RNAase. Three to five replicates were analyzed for every sample, and for each replicate, 20,000 nuclei were measured using a BD FACSCalibur (USA) flow cytometer, which had equipped with a 15 mW, 488 nm argon ion laser. The results of flow cytometry were further analyzed by using the Cellquest software (BD Biosciences). To ensure reliability and repeatability of the obtained results, 3-13 replicates of genome size estimates were performed per species (**Table S4**). The mean genome size per species was used in subsequent data analyses (**Tables 1**, **S1**).

### Models of trait evolution

Phylogenetic signal, evolutionary mode and tempo values of the genome sizes and environmental traits across the phylogeny were investigated in this study. Pagel’s lambda (λ), kappa (κ) and delta (δ) were estimated using the *geiger* (Harmon et al., 2008) and *phytools* (Revell, 2012) packages in R version 4.1.0 (Team, 2013). Pagel’s parameters involving different branch length transformations were used for exploring evolutionary trend of a given trait. The parameter λ indicates different effects of phylogenetic signals of biological traits, κ is related to gradualism or punctuated evolution models, and δ represents evolutionary tempo through the history (Pagel, 1997; Pagel, 1999). We selected λ parameter to test phylogenetic signal due to its better performance than the other indices (Münkemüller et al., 2012).

Brownian motion (BM) and Ornstein-Uhlenbeck (OU) models were popular for the trait evolution (Blomberg et al., 2020). We compared the fitness of six models, including BM, OU, No-signal (λ forced = 0) and Pagel’s three models (λ, δ and κ), through ML estimations in the R package *geiger* (Harmon et al., 2008). The No-signal model forced the λ value to zero, namely, the hypothesis of no phylogenetic signal during the evolution of traits. We used log likelihood and Akaike information criterion (AIC) (Akaike, 1974; Hurvich and Tsai, 1989) values as the metrics for our model performance. Likelihood ratio tests (LRTs) (Vuong, 1989) were used to select the best fitting model for each variable. BayesTraits version 3.0.5 (Pagel and Meade, 2004) was used to verify the ML results of model testing and evaluate whether our traits were suitable for random-walk (model A) or directional model (model B). Bayesian MCMC approach was used to calculate the expected and observed values in different models. In a MCMC analysis, we estimated the log marginal likelihood using 100 stones and 10,000 iterations per stone. We selected the best fitting model depending on Log Bayes Factors (Log BF), which were calculated from the log marginal likelihood values between two models. In order to trace the evolutionary histories of climatic niches in the genus *Eragrostis*, we reconstructed the ancestral states of selected climatic niches using the Mesquite 3.6 (Maddison and Maddison, 2019) with parsimony method.

### Environmental data

The occurrence of each of the *Eragrostis* species under study were extracted from the Global Biodiversity Information Facility (GBIF, https://doi.org/10.15468/dl.y3uyqm). We filtered out erroneous data points (for example, points from the sea) using *CoordinateCleaner* package in R. The resulting distribution data set contained 99,334 localities, with records larger than 150 data points per species. Only two species (*E. humidicola* and *E. acutiglumis*) had narrow range, which were represented by few localities fewer than 50 records. We calculated the medium values of latitudes, longitudes and elevations recorded in the GBIF. These data were then used to calculate the latitudinal and longitudinal ranges.

We obtained 19 bioclimatic variables from the WorldClim 2.1 climate database based on the filtered records (https://www.worldclim.org/data/worldclim21.html) (Fick and Hijmans, 2017). We chose the resolution of 5 mins according to the Du et al. (2017). The data matrix of these 19 bioclimatic niche variables was a summary of the medium value of several climatic dimensions in precipitation and temperature. In addition to climatic niches, we incorporated four climatic niche breadths (Liu et al., 2020) as following: WLNBT, the mean value of the difference between max temperature (warmest month) and min temperature (coldest month) for each locality of a species; SNBT, the differences between max temperatures (warmest month) and min temperatures (coldest month) across all localities of a species; WLNBP, the mean value of the difference between precipitation (wettest-quarter) and precipitation (driest-quarter) for each locality of a species; SNBP, the differences between precipitations (wettest-quarter) and precipitations (driest-quarter) for all localities of a species. Arid environments can be divided into three types based on annual precipitation value: arid zone (< 800 mm), hyper-arid zone (< 300 mm) and semi-arid zone (300-800 mm) (Salem, 1989). In this study, we classified species distributed in arid zones as arid species and others as non-arid species. An overview map of arid species distribution was generated in software ArcGIS 10.8 (ESRI, Redlands, CA, USA).

### Regression analyses

We evaluated the relationships between the genome sizes and environmental factors using phylogenetic generalized least-squares (PGLS) approach in R package *caper* (Orme et al., 2013) and *ape* (Paradis and Schliep, 2019). PGLS model incorporating phylogenetic signal (λ) into matrix, making the regression models more reliable. In PGLS model, λ parameter was adjusted to the optimized value through maximum likelihood method, while κ and δ value were set to one (Revell, 2010). To investigate the issue of whether a more restricted model is better than the less restricted model, the AIC value and LRTs were used to compare the three regression models: non-phylogenetic least-squares analysis (OLS, λ = 0), phylogenetic independent contrasts analyses (PIC, λ = 1) (Felsenstein, 1985; Blomberg et al., 2012) and PGLS (λ = ML). The significant related environmental factors are presented in the scatter plots with *p* value and correlation coefficients.

## Results

### Phylogenetic relationships

**Figure 1:**
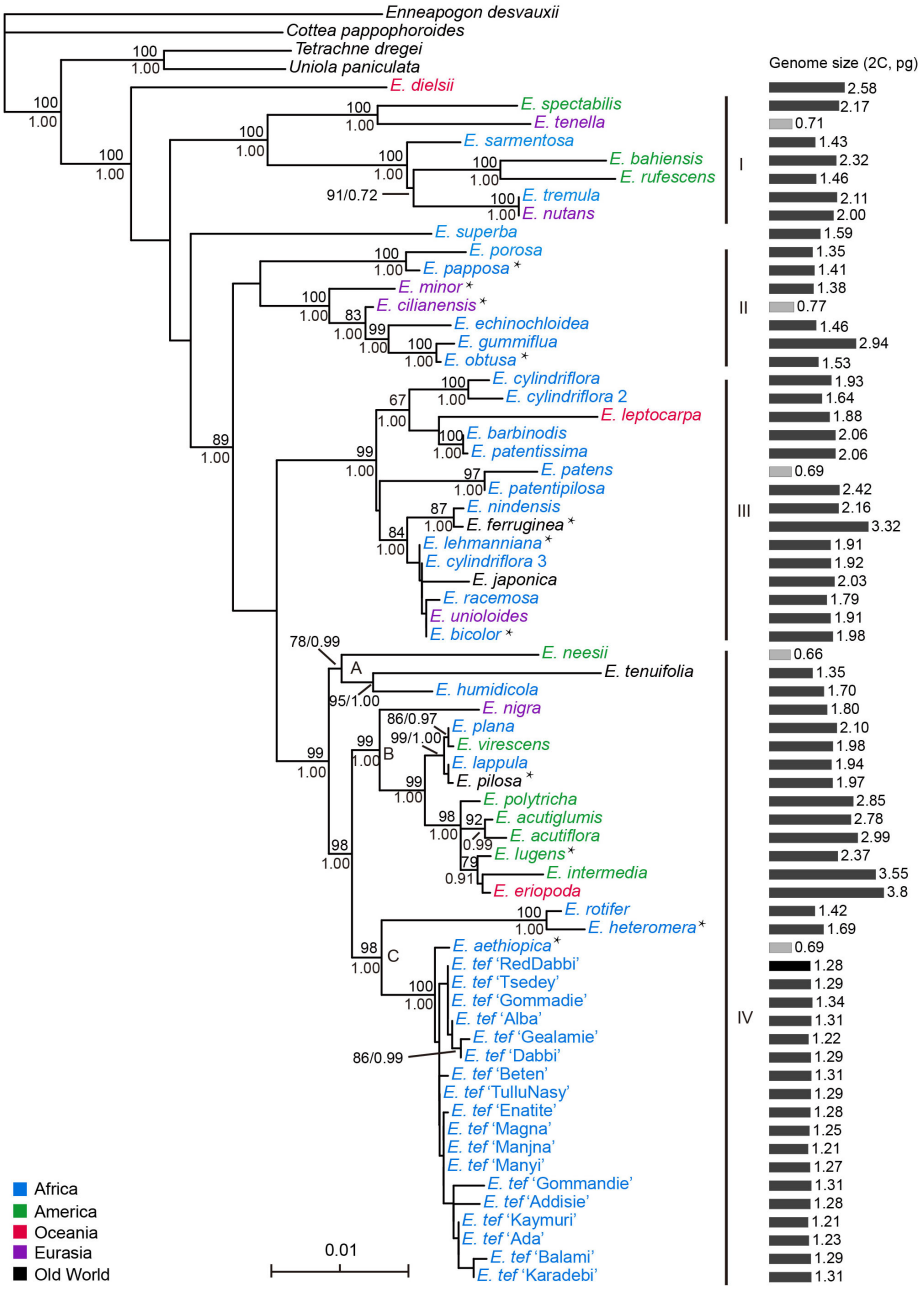
Phylogenetic relationships and genome sizes of *Eragrostis* species. Phylogram of best maximum likelihood tree reconstructed by combined plastid and nuclear ITS data. Numbers above branches are BS values larger than 50%. Numbers below branches are PP values of the Bayesian tree. Taxon color in *Eragrostis* clade corresponding to the centralized distribution area as follows: Africa = blue, America (North America and South America) = green, Australia (or Oceania) = red, Eurasia = purple, Old World = black. “Old World” refers to Europe, Africa, and Asia. Genome sizes (2C, pg) of *Eragrostis* species are presented in grey bars on the right side with small genomes indicated in light grey. *, asterisks represent presumed ancestors of *Eragrostis tef* in Girma et al. (2018).

We tested incongruence length differences (ILD) before we combined ITS and plastid data. The *p* value for ILD was not less than 0.01, therefore, there may be no significant incongruence between ITS and plastid data. Additionally, we reconstructed phylogenies using ITS and plastid data, separately (see **Figures S1**, **S2**). The resulting average lengths of the ITS and plastid data were 757 and 3,010 characters, respectively, of which 49 and 93 characters were variable, and 30 and 43 were informative sites. The results showed relatively low bootstrap values for plastid and ITS trees, respectively, and we thus combined the plastid and ITS data for the subsequent analyses. The aligned data matrix for all samples contained 66 sequences and 3,813 characters. Our results showed that the Bayesian tree was identical to the ML strict consensus tree (**Figure 1**).

We next compared the clades with high bootstrap values among these three trees (ITS, plastid and ITS + plastid). These three phylogenies revealed small discordance. Plastid and ITS trees together supported the monophyly of *Eragrostis* and the basal position of *E. dielsii*. Species of Clade I also formed a strongly supported clade (BS = 100) in plastid tree. However, the two sister clades in Clade I were separated in the ITS tree. The species of large subclade (BS = 100, PP = 1.00) in Clade II, except for *E. minor* in plastid tree, were all formed a single clade in both ITS and plastid trees. Subclade (BS = 84, PP = 1.00) of Clade III were also formed a monophyly in ITS and plastid trees. *E. tef* cultivars and *E. aethiopica* were grouped together in both ITS and plastid trees, which is similar to the Clade IV in **Figure 1**. The only difference is the two species of *E. heteromera* and *E. rotifer* were nested within *E. tef* cultivars in plastid tree.

The monophyly of *Eragrostis* was strongly supported by BS value of 100%, and PP value of 1.00. The Australia endemic species *E. dielsii*, which had a controversial position in *Eragrostis* phylogeny, became the basal species in this clade. The core *Eragrostis* can be further divided into the four clades, termed Clade I-IV (**Figure 1**). After the divergence of *E. dielsii*, the Clade I diverged at first, followed by the Clade II, and finally the Clade III and IV appeared simultaneously. Species included in these clades come from different continents, which were marked in different colors to represent their concentrated area (**Figure 1**). These four clades were supported with BS values more than 98% except the Clade II (BS = 48%, PP = 0.93). Clade I comprised species from America, Africa and Eurasia was strongly supported (BS = 100%, PP = 1.00). The African native species *E. superba* was sister to the Clade II-IV. Two well supported subclades were resolved in Clade II, mainly from South/North Africa, comprising four suggested ancestors (*E. papposa*, *E. minor*, *E. cilianensis* and *E. obtusa*) of *E. tef* (Jones et al., 1978; Girma et al., 2018). They were found to be polyphyletic within this clade. The Clade III consisted of two subclades, one of which was moderately supportive (BP = 67%, PP = 1.00). Eurasian *E. ferruginea* and African *E. lehmanniana* in subclade (BP = 84%, PP = 1.00) of Clade III, had also been identified as the closely relatives of *E. tef* (Girma et al., 2018). Clade IV (BS = 99%, PP = 1.00) diverged into three subclades (IV-A, IV-B and IV-C), with moderate to strong BS values, comprising 18 accessions of *E. tef* and its presumed ancestors (*E. lugens*, *E. heteromera* and *E. aethiopica*) proposed by Jones et al. (1978); Costanza et al. (1979); Bekele and Lester (1981) and Girma et al. (2018). Three species of *E. neesii*, *E. tenuifolia* and *E. humidicola* formed a sister clade (IV-A; BS = 78%, PP = 0.99) to the IV-B and IV-C. In subclade IV-B (BS = 99, PP = 1.00), two species of *E. lugens* and *E. intermedia* belonged to the intermedia complex in the early taxonomic period (Witherspoon, 1975), had close relationship in our study. Previous studies suggested that the *E. pilosa* was close to *E. tef* (Ayele et al., 1999; Ayele and Nguyen, 2000; Ingram and Doyle, 2003), but *E. pilosa* and teff cultivars were polyphyletic in this study. Different sampling could possibly explain this discrepancy. Our results showed that *E. aethiopica*, *E. heteromera* and *E. rotifer* from Africa were most closely related to *E. tef* in the subclade IV-C (BS = 98%, PP = 1.00). Among them, *E. aethiopica* and *E. heteromera* were proposed as ancestors of *E. tef* (Girma et al., 2018).

The four clades were partially supported by the blade anatomy analyses of the *Eragrostis* species, according to the previously defined types of NAD-ME-like, PCK-like, and Intermediate (Ellis, 1977; Prendergast et al., 1986; Ingram, 2010). Clade I was characterized by the PCK-like type, except *E. spectabilis* and *E. rufescens* (without anatomy information). The anatomical type NAD-ME-like was randomly dispersed in the other three clades. Interestingly, the presumed progenitors of *E. tef* mentioned above were dispersed into different clades rather than forming a single clade, whereas they shared a similar blade type of Intermediate, except *E. papposa* and *E. obtusa* with NAD-ME-like type.

### Genome size variation

**Figure 2:**
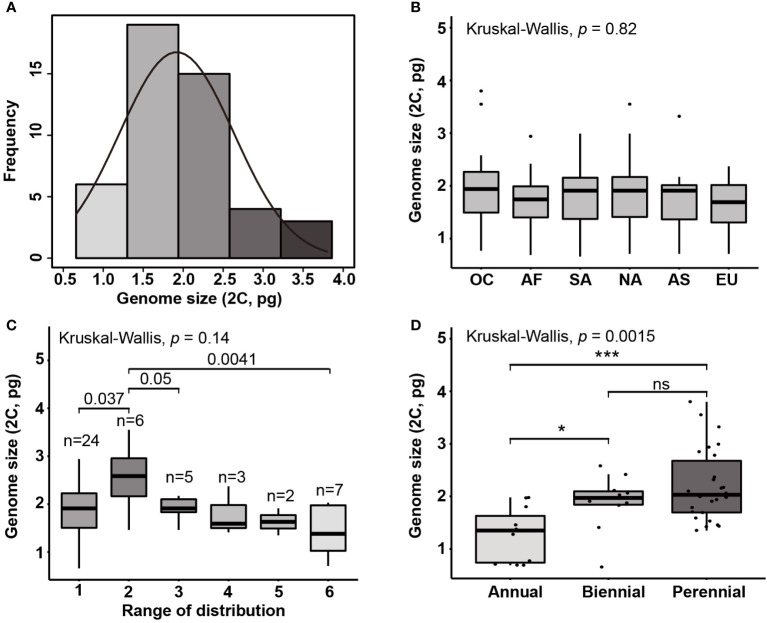
**(A)** Histogram of genome sizes (2C; pg) in *Eragrostis* and the normal density curve. Boxplots show the genome size variation on **(B)** six continents **(C)** range of distribution (occupied continents) and **(D)** life styles. The value of occupied continents corresponding to species occurred in one continent to six continents, according to the distribution data from GBIF. OC, Oceania; AF, Africa; SA, South America; NA, North America; AS, Asia; EU, Europe. Ns, not statistically significant; ***, *p* < 0.001; *, *p* < 0.05.

The genome size estimates of 66 *Eragrostis* accessions representing globally geographical origins were reported in this study. The chromosome numbers and DNA contents formerly reported were also collected and given in **Table 1** and **Table S1**. Our results showed that genome sizes differed up to 6-fold, ranging from 0.66 pg in *E. neesii* to 3.8 pg in *E. eriopoda* (**Figure 2A**). The five species (*E. tenella*, *E. cilianensis*, *E. patens*, *E. neesii* and *E. aethiopica*) with the smallest 2C-value (0.66-0.77 pg) were half that of *E. tef*, indicating that they might be diploid rather than tetraploid, supporting previous chromosome counting with 2n = 2x = 20 (Dujardin and Tilquin, 1971; Fernandes, 1971; Bir, 1983; Gohil, 1986; Tavassoli, 1986; Faruqi et al., 1987). The three species with the largest genomes were *E. ferruginea*, *E. intermedia* and *E. eriopoda* (3.32-3.80 pg), showing that they might be decaploid or with even higher ploidy levels, confirmed by previous chromosome counting with 2n=12x=120 (Tateoka and Löve, 1967; Gould, 1968; Reeder, 1971) (**Table 1**). Among the 11 presumed progenitors of *E. tef* (Girma et al., 2018) (indicated by asterisks in **Figure 1**), genome sizes ranged from 0.69 pg in *E. aethiopica* to 3.32 pg in *E. ferruginea*, and their ploidy levels fluctuated from diploid to decaploid. Although, the genome sizes varied by 4.8-fold in presumed ancestors of *E. tef*, they seemed still within the range of genome size variation of our examined samples (indicated with asterisk; **Table 1**). Additionally, previous studies reported that *E. cilianensis* and *E. lehmanniana* contained multiple cytotypes (Bekele and Lester, 1981; Voigt et al., 1992), but we failed to observe such results in this study. The DNA contents (2C-value) were estimated for the three species of *Eragrostis*, including *E. spectabilis* (2.30 pg) (Bai et al., 2012), *E. minor* (1.46 pg) (Šmarda et al., 2014) and *E. tef* (1.40 pg) (Bennett and Smith, 1976). Our results obtained in this study are quite consistent with the formerly reported genome sizes for these three species (**Table 1**), suggesting the reliability of experimental methodology. Notably, we observed an average genome size of 1.28 pg for the 18 *E. tef* varieties (2n = 4x = 40) (**Table S1**), which was in accordance with the recently published genome sequences of 1.27pg (VanBuren et al., 2020).

Given the morphological variability and wide range of *Eragrostis* species, we analysed the genome size variation among different clades, distribution ranges and life styles. There were no significant differences in genome sizes among the four clades (*p* = 0.284; Kruskal-Wallis test). Within each clade, genome sizes varied limitedly in Clade II (0.71-2.32 pg) and greatest in Clade IV (0.66-3.80 pg). Interestingly, we found that at least one annual species of each clade was small in genome size, which are indicated in light grey colour (0.66-0.77 pg) (**Figure 1**; **Table 1**).

We then tested genome size variation among six continents, as shown by the Kruskal-Wallis test. Our results showed that differences were not statistically significant (*p* = 0.820; **Figure 2B**). We first attempted to only consider the concentrated area for each species, however, some species occurred in more than one continent. Thus, the number of continents occupied by each species was used to represent the ranges of species by following Wang et al. (2016). However, *Eragrostis* is much more widely distributed than *Betula*, and thus, we used the number of continents occupied by species to represent the distribution range. We divided samples into six groups, 1 for one continent and 6 for six continents (**Table S5**). The group occupied six continents had the smaller genome size than the group occupied two continents (Wilcoxon test, *p* = 0.004; **Figure 2C**; **Table S5**). The group occupied two continents also exhibited differences with groups occupied one and three continents (Wilcoxon test, *p* = 0.037, *p* = 0.050, respectively; **Figure 2C**). Moreover, perennial species had larger genome sizes than the annual species (*p* < 0.001; Wilcoxon test) (**Figure 2D**).

### Genome size evolution

The comparisons among the six evolutionary models (see Materials and methods) showed that the kappa model fitted best in genome size with the lowest AIC score (**Table 2**). Estimates of Pagel’s λ indicated that the genome size exhibited a moderate phylogenetic signal with a lambda value of 0.75 (**Table 2**) and LRTs verified the results (**Table S6**). Indeed, we observed that several species with overlapping distributions or genome sizes tended to have close relationships in subclades. In addition, BayesTraits results suggest that λ value of genome size was significantly greater than zero but lower than one (**Table S7**), indicating that genome size failed to evolve under a pure Brownian motion. Environmental factors may also play an important role in determining genome sizes of *Eragrostis*. The estimates of Pagel’s δ was 3.00, which was significantly different from 1 (**Table S7**), referring to the occurrence of specific adaptation. Estimates of the Pagel’s κ value revealed that short branches contributed proportionally more to the genome size evolution (κ = 0.43; **Table 2**). Although genome size had a strong phylogenetic signal, the λ value was still significantly different from 1 (**Table S7**), indicating that genome size was not very suitable for either pure Brownian motion (λ = 1) or No-signal model (λ = 0). BM and OU are basic models used in continuous variables, and thus, we compared the log likelihood of the basic models through LRTs. The results indicated that the restricted model (BM) fitted the data better than the less restricted model (OU), because we accepted the H0 hypothesis that assumes the restrictions are ‘true’ (*p* = 0.334). Hence, it is likely that the genome size evolved towards a BM model through the history. Besides, the Log BF of the model B was not significantly different from the model A, confirming that the genome size fitted the random walk better than the evolution of directional trend (**Table S7**).

**Table 2:** Selection for the best evolutionary model of genome size and environmental factors in the genus *Eragrostis*.

Model	Parameter	AICc	Lh	*k*
Genome size				
BM		144.56	-70.15	2
Lambda	λ = 0.75	101.85	-47.66	3
Delta	δ = 2.99	132.04	-62.75	3
**Kappa**	**κ = 0.43**	**98.69**	**-46.08**	**3**
No-signal	λ forced = 0.00	106.77	-51.26	2
OU	α = 2.72	145.9	-69.68	3
Annual Precipitation				
BM		1028.39	-512.19	2
Lambda	λ = 0.00	715.89	-354.95	3
Delta	δ = 2.99	1019.19	-506.60	3
Kappa	κ = 0.00	726.75	-360.38	3
**No-signal**	**λ forced = 0.00**	**714.17**	**-354.95**	**2**
OU	α = 2.72	1029.34	-511.67	3
Mean Diurnal Range				
BM		536.15	-265.94	2
Lambda	λ = 0.00	223.97	-108.70	3
Delta	δ = 2.99	527.23	-260.34	3
Kappa	κ = 0.00	244.64	-119.04	3
**No-signal**	**λ forced = 0.00**	**221.68**	**-108.70**	**2**
OU	α = 2.72	537.39	-265.42	3
SNBT				
BM		575.62	-285.67	2
Lambda	λ = 0.00	380.94	-187.19	3
Delta	δ = 2.99	569.34	-281.39	3
Kappa	κ = 0.00	390.58	-192.01	3
**No-signal**	**λ forced = 0.00**	**378.66**	**-187.19**	**2**
OU	α = 2.72	576.86	-285.15	3
Latitude				
BM		660.84	-328.29	2
Lambda	λ = 0.00	450.93	-222.19	3
Delta	δ = 2.89	659.99	-326.72	3
Kappa	κ = 0.00	469.51	-231.47	3
**No-signal**	**λ forced = 0.00**	**448.65**	**-222.19**	**2**
OU	α = 2.72	662.09	-327.77	3

The selected models were indicated in bold; BM, Brownian motion; OU, Ornstein-Uhlenbeck; No-signal (λ forced = 0) model; k, free parameters; Lh, log likelihood; AICc, corrected AIC value.

As for geographical factors (latitude, longitude and elevation), the model that best fitted the data was No-signal model (λ forced = 0.00; all *p* > 0.05 between the λ and No-signal model; **Tables 2**, **S6**, **S8**) based on the AICc criterion, indicating a lack of phylogenetic signals in these geographical variables. The LRTs between basic models did not achieve significance at the 0.05 level, suggesting that BM models performed better than OU models in all geographical factors (*p* = 0.307-0.320; **Table S6**). The Log BF provided strong evidence that the δ values for geographical factors were all greater than 1 (δ = 2.89-2.99; **Tables 2**, **S7**, **S8**), indicating that there was either a temporally latter trait evolution or an accelerated evolution over time. These three geographical variables were consistent with the punctuated evolution (κ = 0.00; **Tables 2**, **S8**), which was supported by both ML and MCMC methods (**Table S7**).

Evolutionary patterns of all climatic niches were similar to geographical variables to fit a No-signal model (**Tables 2**, **S8**) and a high value of δ (δ = 2.99) (**Tables 2**, **S8**). Estimated values of κ were generally low and significantly different from one in all climatic niches (**Tables 2**, **S7**, **S8**). The lambda, kappa, and delta models clearly outperformed the BM models except that four variables (temperature annual range, latitude, SNBP and WLNBT) showed no significant differences between the delta and BM models (**Tables 2**, **S6**, **S8**). The OU models had never shown any significant differences from the BM models based on the LRTs in all climatic niches (**Table S6**).

### Correlations between genome sizes and environmental factors

Exploring the correlations between genome sizes and environmental factors was one way to offer evidence for the evolutionary adaptation of plants (Petrov, 2001; Šmarda et al., 2014; Bilinski et al., 2018). The results of regression analyses between genome sizes and environmental factors used in three models are presented (**Tables 3**, **S9**; **Figure S3**). Overall, there were no correlations between the genome sizes and the environmental factors (with the exception of eastern longitude) in ordinary regression models, whereas after phylogeny correction, significant genome size variation in *Eragrostis* could be explained by eight factors in the PGLS models (**Table 3**). In fact, trait evolution usually depends on phylogeny, and the PGLS approach incorporates the value of phylogenetic signal as covariance into the regression analyses, giving a better way to handle our data. Indeed, LRTs in all variables showed that the PGLS models fitted our data better than OLS significantly (all *p* < 0.001; **Tables 3**, **S9**). The PIC model assumed that the traits of closely related species evolved independently under the Brownian motion. Although some correlations were not as strong as expected in a strict Brownian model, LRTs chose PGLS regression as the best fitting model, i.e. precipitation of wettest month showed high correlation with genome size in PIC model, but LRTs chose PGLS model with no correlation (**Table S9**).

**Table 3:** Summary of associations between *Eragrostis* genome sizes (2C, pg) and environmental factors tested by three regression models.

Model	*R^2^*	*b*	*p*	AIC	Lh	LRTs
Mean Diurnal Range-genome size						
**PGLS**	**0.546**	**0.065**	**<0.001**	**43.72**	**-19.86**	
OLS	0.022	0.298	0.318	52.40	-24.20	<0.001
**PIC**	**0.546**	**0.065**	**<0.001**	**43.72**	**-19.86**	−
Mean Temperature of Driest Quarter-genome size						
**PGLS**	**0.105**	**-0.211**	0.026^*^	**43.11**	**-19.55**	
OLS	0.029	-0.124	0.253	52.07	-24.04	<0.001
PIC	0.003	-0.005	0.697	80.63	-38.32	<0.001
Annual Precipitation-genome size						
**PGLS**	**0.541**	**-0.027**	**<0.001**	**44.17**	**-20.09**	
OLS	~0.000	-0.018	0.877	53.43	-24.72	<0.001
**PIC**	**0.541**	**-0.027**	**<0.001**	**44.17**	**-20.09**	−
Precipitation of Warmest Quarter-genome size						
**PGLS**	**0.565**	**-0.023**	**<0.001**	**41.66**	**-18.83**	
OLS	0.003	0.04	0.697	53.30	-24.65	<0.001
**PIC**	**0.565**	**-0.023**	**<0.001**	**41.66**	**-18.83**	−
Precipitation of Coldest Quarter-genome size						
**PGLS**	**0.562**	**-0.015**	**<0.001**	**41.96**	**-18.98**	
OLS	~0.000	0.006	0.916	53.44	-24.72	<0.001
**PIC**	**0.562**	**-0.015**	**<0.001**	**41.96**	**-18.98**	−
Northern Latitude-genome size						
**PGLS**	**0.834**	**-0.111**	**<0.001**	**13.14**	**-4.57**	
OLS	0.050	-0.172	0.409	22.29	-9.14	<0.001
**PIC**	**0.834**	**-0.111**	**< 0.001**	**13.14**	**-4.57**	−
Eastern Longitude-genome size						
**PGLS**	**0.166**	**0.130**	**0.014**	**31.60**	**-13.80**	
**OLS**	**0.166**	**0.130**	**0.014**	**31.60**	**-13.80**	−
PIC	0.293	-0.023	<0.001	44.71	-20.35	<0.001
SNBT-genome size						
**PGLS**	**0.503**	**-0.172**	**<0.001**	**47.94**	**-21.97**	
OLS	0.027	-0.255	0.267	52.16	-24.08	<0.001
**PIC**	**0.503**	**-0.172**	**<0.001**	**47.94**	**-21.97**	−

The best fitting models are indicated in bold. b, slope; AIC, Akaike information criterion; Lh, log likelihood; LRTs, likelihood ratio tests.

There were significant negative relationships between genome sizes and the three climatic niches associated with precipitation, including annual precipitation, precipitation of warmest quarter and precipitation of coldest quarter (*R^2^* = 0.541, *R^2^* = 0.565 and *R^2^* = 0.562, respectively; *p* < 0.001; **Table 3**). For temperature niches, genome size was significantly positively associated with the mean diurnal range (*R^2^* = 0.546, *p* < 0.001), and negatively correlated with SNBT (*R^2^* = 0.503, *p* < 0.001) and the mean temperature of driest quarter (*R^2^* = 0.105, *p* < 0.05) (**Table 3**). The relationships between genome sizes and geographical distributions were also investigated. There was a slightly significant positive relationship between the genome size and eastern longitude (*R^2^* = 0.166, *p* < 0.05; **Table 3**), which was the only exception when the OLS model fitted the data better than the PIC model. In contrast, a significant negative relationship was observed between genome size and northern latitude in the PGLS model (*R^2^* = 0.834, *p* < 0.001; **Table 3**). However, the correlation coefficient in the northern latitude was probably unreliable due to the small sample distributions in the northern hemisphere. In these eight correlated variables, the PIC regression models gave similar results to those of the PGLS, except for the mean temperature of driest quarter and the eastern longitude, showing that the assumed Brownian motion in the PIC models was correct (Blomberg et al., 2012).

### Climatic niches, ancestral states and global distribution

**Figure 3:**
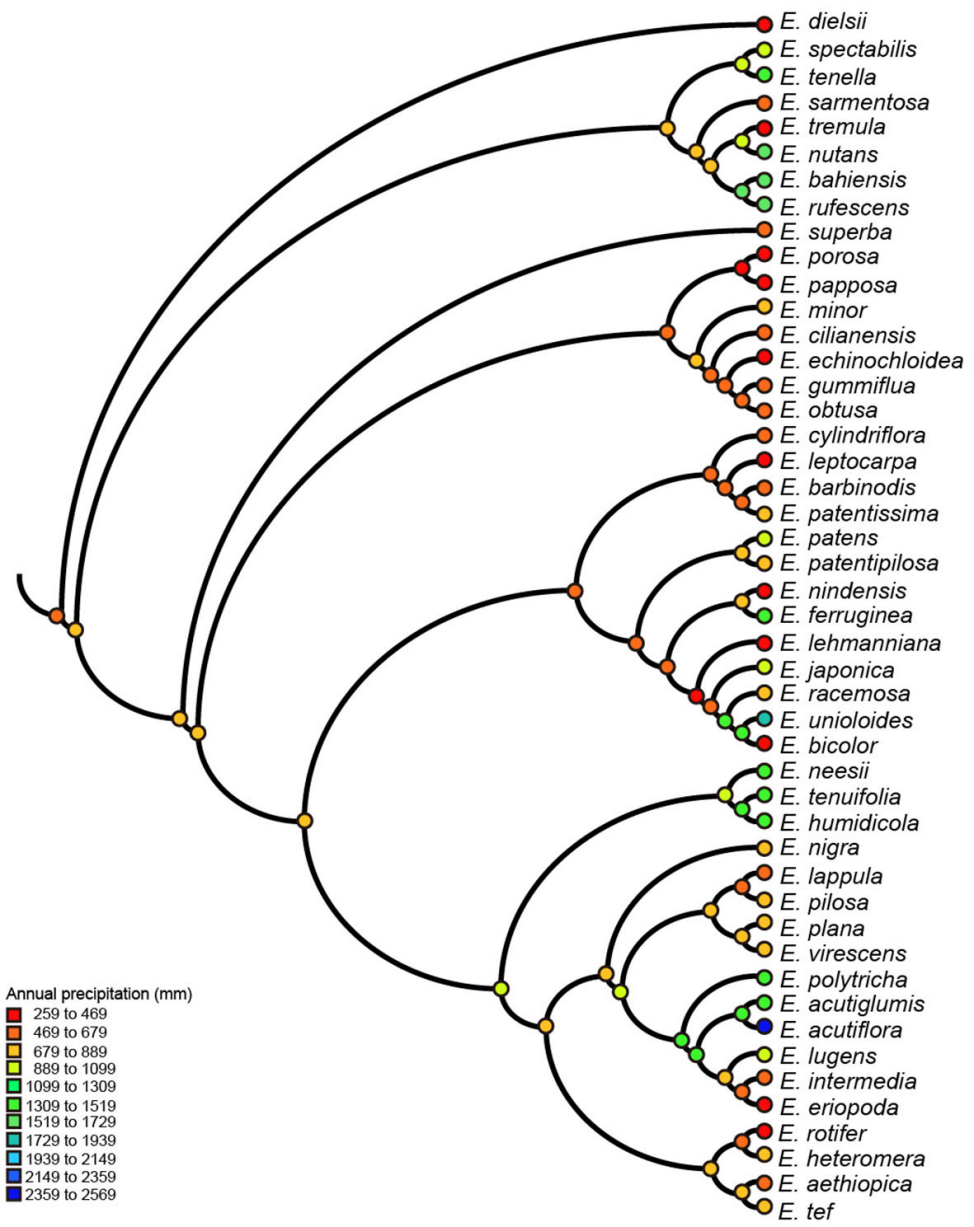
Parsimony reconstruction of ancestral annual precipitation (mm) on *Eragrostis* consensus tree (balls & sticks). Different values of precipitation were marked as different colors.

We reconstructed ancestral character states only for SNBT, mean diurnal range and annual precipitation, all of which had strong evolutionary associations with genome sizes. Ancestral states were inferred using the maximum parsimony method for all nodes of the phylogenetic tree, but only some key nodes are presented in **Figure S4** (labeled with numbers). The results of ancestral reconstruction showed that the common ancestors of the *Eragrostis* clade came from semi-arid areas (~648 mm of annual precipitation) with the mean diurnal range of approximately 13°C. Climatic niche values of ancestral nodes were estimated between 596 mm to 928 mm for precipitation, and between 12°C to 15°C for the mean diurnal range. In the terminal taxa, *E. dielsii*, the most basal species in the clade *Eragrostis* showed the lowest annual precipitation at only 259 mm, and *E. acutiflora* in Clade IV showed a maximum value of 2,359 mm. The diurnal range value varied from 7°C in *E. nutans* to 16°C in *E. lehmanniana* (**Figures 3**, **S4**). Thus, the climatic conditions in the reconstructed ancestral nodes were much more stable than the extant species. Besides, approximately 50% of extant species with annual precipitation lower than 800 mm were distributed across the phylogenetic tree for each clade (**Figure 3**). Indeed, the Wilcoxon tests showed that there was no difference in annual precipitation between the clades (*p* > 0.05), except for the Clade II (*p* = 0.039), by comparing each group with the base value. Within a particular Clade II, all species had low precipitation and remained relatively stable to basal nodes (**Figures 3**, **S4**). Interestingly, most of the key nodes corresponding to the ancestors of two clades had intermediate values between them. This can be observed, for example, the Node-1 value was estimated at 701 mm of annual precipitation, which was between 660 mm (Node-9) and 764 mm (Node-5). For SNBT, the ancestral status of this genus was estimated to be 49°C (Node-1), with a slow increase during the evolution towards Clade II (Node-9, 56°C) and a gradual decrease during the evolution towards Clade IV (Node-6, 43°C). The annual species *E. pilosa* had the greatest niche breadth, from -36°C to 45°C (SNBT = 81°C). Conversely, a perennial species of *E. humidicola*, narrowly inhabited in Central Africa (Senzota, 1982), had the smallest habitat temperature breadth from 9°C to 32°C (SNBT = 23°C) (**Figure S4**). Notably, our estimates of these three ancestral climatic niches revealed signs of both increase and decrease in the four clades (Clade I-IV).

**Figure 4:**
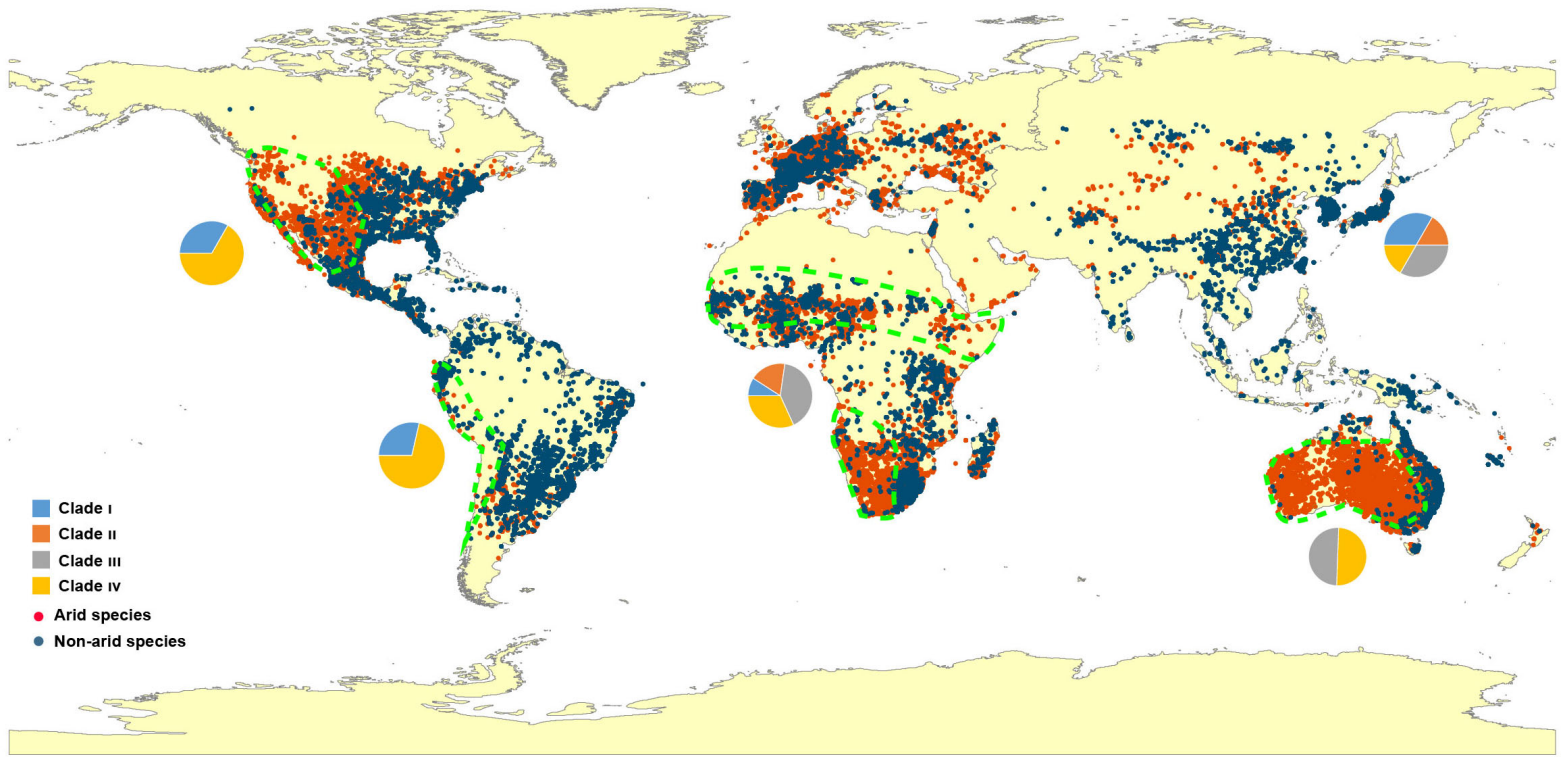
Map of locations and distributions of *Eragrostis* species used in this study. The regions surrounded by the green dashed lines represent the overlapping arid areas according to the arid species and the survey of International Arid Lands Consortium (http://pubs.usgs.gov/gip/deserts/what/world.html). Red dots, arid species; Dark blue dots, Non-arid species. The pie charts indicate the proportion of four major Clade I-IV in each continent. Blue, Clade I; Orange, Clade II; Grey, Clade III; Yellow, Clade IV.

A global distribution map is presented to review the dispersal of *Eragrostis* species (**Figure 4**). Unexpectedly, the arid species (red dots) showed a similar distribution area when compared to the published map of “Arid Lands of the World” (http://pubs.usgs.gov/gip/deserts/what/world.html). South Africa, North America and Australia showed the greatest coincidence with the *Eragrostis* species in the arid areas (areas surrounded by green dashed lines). Arid species inhabited almost all of these three continents and were partially dispersed in North Africa, South America and Eurasia. Of these arid species, *E. aethiopica* endemic to South Africa had the smallest genome size of 0.69 pg, while *E. eriopoda* native to Australia had the largest genome size of 3.80 pg. The four species of *E. tef*, *E. cilianensis*, *E. minor*, and *E. virescens*, had relatively small C-values (0.77-1.98 pg), mainly distributed in semi-arid zones around the world (Northam et al., 1993; Ketema, 1997; Martini and Scholz, 1998; Rajbhandari et al., 2016). In contrast, the three species of *E. dielsii*, *E. leptocarpa* and *E. eriopoda* had relatively large C-values (1.88-3.80 pg), which were endemic to Australia with annual precipitation less than 300 mm.

We finally investigated the genome size variation in 27 arid species across Africa, America and Australia. Perennial species had a higher percentage in the arid zones of these three continents (**Figure S5A**). However, genome sizes of arid species showed no differences between these three fully occupied arid areas (*p* = 0.610; **Figure S5B**). When we divided all species into arid and non-arid groups, the significance of the differences was not confirmed by the Wilcoxon test (**Figure S5C**), even among different life style groups (**Figure S5D**).

## Discussion

### Phylogenetic relationships and genome size variation

Previous studies have put efforts to investigate the monophyly of *Eragrostis*, but until recently, it is still a controversial issue. Our results strongly support the monophyletic origin of *Eragrostis*, which is in accordance with the phylogeny analyses of combined *rps16* and *trnL-F* (Ingram, 2010) and complete chloroplast genomes (Somaratne et al., 2019). Nevertheless, other researchers proposed opposing opinions that they have reintegrated some previously separated species into *Eragrostis* (Hilu and Alice, 2001; Ingram and Doyle, 2004; Ingram and Doyle, 2007; Peterson et al., 2010). Different and limited sampling may be responsible for these discrepancies, and thus the origin of this large genus still needs further investigation.

The infrageneric classification of *Eragrostis* has been complicated due to the occurrence of polyploids, and only a few studies have resolved some close relationships (Ingram and Doyle, 2007; Ingram, 2010; Girma et al., 2018; Barrett et al., 2020; Liu et al., 2021). The *E. dielsii* growing in a drier area of Australia with precipitation less than 300 mm annual year was the basal species of the *Eragrostis* clade in this study. This finding is consistent with former results by Ingram and Doyle (2007), in their *waxy* and *rps16* tree, by Girma et al. (2018), in their new *waxy* gene tree, by Peterson et al. (2010), in their nuclear ITS sequence analyses, and by Barrett et al. (2020), in their combined plastid (*rpl32-trnL*, *rps16*, *rps16-trnK*) and ITS analyses. A revision of *Eragrostis* in Australia mentioned that *E. dielsii* was also a salt-tolerant plant (Lazarides, 1997). Thus, due to its taxonomic status and its tolerance to aridity, further efforts are required to explore its important role in *Eragrostis*. Additionally, another important species of *Eragrostis* was *E. tef*, whose ancestors are still unknown. The combined data and plastid data alone (**Figure S1**) together support that *E. aethiopica*, *E. heteromera* and *E. rotifer* were closely related to *E. tef*. To identify the diploid teff donors (Ingram and Doyle, 2003; Girma et al., 2018; VanBuren et al., 2020), however, a well-resolved phylogeny based on high-quality sequences is still needed, which is critical in exploring the complex evolutionary history of this allopolyploid crop.

Genome size variation has long been linked to life styles and ranges, while we are still wondering whether this link is stable. If so, it could be possible to choose the area in which a plant would grow best (Bennett, 1987). It is well known that genome size variation has effects on distribution ranges of species (Knight and Ackerly, 2002; Knight et al., 2005). This was observed in the genus *Eragrostis*, supporting the hypothesis that species with smaller genomes occupy a wider area than the species with larger genomes (Grotkopp et al., 2004; Lavergne et al., 2010; Pandit et al., 2014). Invasive ability of the species with small genomes, such as *E. tenella*, *E. minor* and *E. curvula*, which were reported as invasive plant species (Guido et al., 2019; Roberts et al., 2021; Wróbel et al., 2021; Buerdsell et al., 2022), could explain their global dispersal (Bennett et al., 1998; Grotkopp et al., 2004). Overall, our study suggests that genome size variation in *Eragrostis* is one of important drivers affecting global distribution, and species with smaller genome sizes tend to have a more extensive distribution than those of larger sizes.

The genome size of land plants has generally shown a non-normal or skewed distribution (Leitch and Leitch, 2013). In contrast, the genome size of *Eragrostis* in this study was subjected to normal distribution with large and small genomes in equivalent numbers (**Figure 2A**). Normal distribution of genome size was also found in six continents, and species with the small genome size occurred in each clade and continent. Besides, genome size variation showed no difference among continents and clades. Hence, most continents or phylogenetic clades might follow a similar pattern of genome size variation. It is interesting to note that this kind of trend, without differences between continents, has also been documented in other worldwide species like *Chenopodium* (Mandak et al., 2016), duckweeds (Wang et al., 2011) and *Carex* (Chung et al., 2011). Our results indicate that small genome size favored annual species. This is consistent with previous studies, which claimed that annuals or ephemerals were either rare or absent from those very large amounts of DNA (Price and Bachmann, 1975; Bennett, 1987; Enke et al., 2011; Qiu et al., 2019).

### Evolutionary dynamics

The genome size variation in *Eragrostis* was found to have a moderate phylogenetic signal, indicating that the genome size evolution may be associated with the phylogenetic relationships. Many species have shown strong phylogenetic signals, which may be related to the heritable characteristic of genome size (de Ricqlès et al., 2008). The presence of a complete phylogenetic signal, a value λ approaching one, suggests that genome size did not evolve in an adaptive fashion (Blomberg et al., 2003; Pandit et al., 2014). However, the LRTs results showed that the phylogenetic signal was significantly different from 1, indicating the occurrence of adaptive evolution in *Eragrostis* species (Gregory, 2005; Gregory and Johnston, 2008). This observation was also supported by the results of our PGLS analyses, showing that genome size was significantly related to climatic variables. Furthermore, climatic niches and geographical factors showed similar λ model with lacking of phylogenetic signal, indicating that PNC did not exist (Losos, 2008). The likely explanation is that existing species were less likely to maintain their ancestral environmental features and adapted to new niches (Wiens and Graham, 2005). These evolutionary models were further confirmed by the ancestral states of the climatic niches. Annual precipitation showed that the ancestral states for *Eragrostis* has undergone several reductions and increases along different clades, as well as the expansions of SNBT and mean diurnal range, indicating that existing species had a larger area than their ancestors did.

Evolution often breaks down or bursts out under punctuated model (Eldredge, 1993; Gregory, 2004; Pagel et al., 2006). Our results suggest punctuated environmental changes, and the genome size of *Eragrostis* evolved in more punctuated than graduated behaviors. This finding is in good agreement with Elena et al. (1996), who claimed that evolution went punctuated when environmental changes became erratic. Punctuated evolution of genome size has been reported in some other plants, such as *Melanocrommyum* (Gurushidze et al., 2012), Liliaceae (Leitch et al., 2007), *Orobanche* (Weiss-Schneeweiss et al., 2006), and bromeliads (Müller et al., 2019). However, the drastic genome size variation of *Eragrostis* may also be a result of whole genome duplication, as multiple ploidy levels have been reported in this genus (Tavassoli, 1986).

Tempo estimates of all variables, including genome sizes and environmental factors, gave consistent results with δ values from 2.69 to 2.99. All variables may have experienced species-specific adaptation or accelerated evolution over time, with more diversification during recent evolutionary history, as seen in other taxa (Kang et al., 2014; Müller et al., 2019; Martínez-Méndez et al., 2019). In *Eragrostis*, most arid species from the recently diversified clades could explain the latter evolution model. However, the cause of this tendency still remains unsolved.

### Environmental correlation and global dispersal

Genome size variation associated with diverse environmental factors have often been interpreted as a kind of adaptation in response to the precipitation or temperature changes (Bennett, 1987; Wakamiya et al., 1993; Petrov, 2001; Whitney et al., 2010; Kang et al., 2014; Jordan et al., 2015; Trávníček et al., 2019). Previous opinions about plant species with large genome sizes remain controversial. It was suggested that the extra DNA content would have maladaptive consequences and tend to be excluded from the extreme environments (e.g. high temperature, low precipitation) (Knight and Ackerly, 2002; Knight et al., 2005). In this study, however, we found the contrary evidence. Our PGLS results suggest that genome sizes appeared to have strong correlations to precipitation related niches but nothing was related to precipitation seasonality. Specifically, species with larger genomes preferred less precipitation than the species with smaller genomes. Not coincidentally, similar trends of larger genomes in arid areas were also reported (Kalendar et al., 2000; Torrell and Vallès, 2001; Bureš et al., 2004; Grotkopp et al., 2004; Souza et al., 2019). Considering that ploidy levels are great drivers of genome sizes in the genus *Eragrostis*, one likely interpretation is that large genomes indeed represent plant species with higher ploidy levels, which possess superior phenotypes, may be better adapted to extreme environmental conditions. In addition, plant species with large genomes have been proposed to survive in extreme habitats through cell inflation instead of cell division (Grime and Mowforth, 1982).

Although the correlation coefficients in the temperature niches were smaller than the precipitation-related niches, the mean diurnal range also showed a close relationship with genome size in *Eragrostis*. The mean diurnal range was considered as a climatic risk factor because a major change in diurnal temperature could increase plant mortality (Kan et al., 2007; Briga and Verhulst, 2015). In this study, genome size was positively correlated with the mean diurnal range, indicating that *Eragrostis* species with large genomes, in other words, higher levels of polyploids may be more permissible in habitats where diurnal temperatures vary greatly. Correlations between genome sizes and mean diurnal ranges were also found in wild wheat, *Primulina* and *Ranunculus auricomu* (Özkan et al., 2010; Kang et al., 2014; Paule et al., 2018). It is well known that the climatic niche variables are interdependent, and thus, the relationships among them were further considered in a context of association with genome size variation. An intimate relationship was established between annual precipitation and mean diurnal range with negative significance in the OLS and PGLS models (*R^2 =^* 0.530, *p* < 0.001), suggesting that the genome size variation in *Eragrostis* may result from the combined effects of precipitation and temperature. Additionally, previous studies suggested that genome size variation along geographic gradients was often regarded as adaptive signatures (Rayburn and Auger, 1990; Bottini et al., 2000; Kang et al., 2014; Bilinski et al., 2018; van Boheemen et al., 2019). We observed a positive relationship between genome size and eastern longitude, telling that adaptive evolution may occur in *Eragrostis*. Not surprisingly, this kind of phenomenon has already been reported in *Hieracium*, *Allium* and *Bituminaria bituminosa* (Walker et al., 2006; Chrtek et al., 2009; Duchoslav et al., 2013). Overall, our global analyses highlight a likely role of climatic niches in shaping the genome size evolution of *Eragrostis*. The association of precipitation with the genome size variation may be indicative of the widespread occurrence of *Eragrostis* species in arid area. However, the global dispersal of *Eragrostis* species is probably more complicated than we thought. Other factors such as human activities may have influenced the dispersal of *Eragrostis*.

The ancestors of the extant *Eragrostis* species originated in arid areas, indicating that drought tolerance may already be apparent in ancestral states. While large genomes were correlated with low precipitation, some species with small genomes also occurred in arid zones (e.g. *E. tef*, *E. cilianensis*, *E. minor*, and *E. virescens*). It was reported that species with small seeds and genomes had advantages in terms of plant invasion (Grotkopp et al., 2004; Lavergne et al., 2010). Indeed, *E. cilianensis*, *E. minor* and *E. virescens* have been used as introduced species in the livestock industry in many countries (Martini and Scholz, 1998; Peterson and Giraldo-Canas, 2012). This may be one of the explanations for the global spread of *Eragrostis* species, and their increasing drought tolerance ability on other lands. Furthermore, the absence of PNC in the precipitation niche variables implied that the species departed from the original habitats. The ancestral reconstruction indicated that the existing niches may come from a decrease or increase of the ancestral states in each clade. We thus hypothesize that *Eragrostis* species with large genome sizes or high polyploidy levels may have dispersed into new niches with even drier or wetter conditions in each clade. Repeated adaptation to similar climatic gradients has resulted in the rapid spread of invasive plants (van Boheemen et al., 2019). Therefore, species-specific adaptations to environmental changes might occur several times in *Eragrostis* because of complicated polyploidy history across the global area. This may explain why each clade had a similar pattern of variation for both large and small genomes from different areas. Such results were also observed in the large subfamily Bromelioideae, the most diverse clade of Bromeliaceae (Silvestro et al., 2014; Müller et al., 2019). Considering a large number of *Eragrostis* species are still missing in this study, more sampling is necessary to understand the environmental correlation and the global dispersal in a context of genome size evolution in *Eragrostis*.

To our knowledge, this is the first study to investigate the genome size variation and evolution in the genus *Eragrostis*. Our results suggest that an evolutionary adaptation of genome sizes, which mainly originated from complicated polyploidization history, were associated with precipitation in the genus *Eragrostis* across their global distribution. The significant negative correlation between the genome sizes and precipitations seemingly supports an adaptive consequence of the genome size evolution in *Eragrostis*. The expansion of climatic niches throughout the history and the species-specific adaptation model in genome size further confirm that the large genomes of *Eragrostis* species have evolved as evolutionary products in various environments. In fact, adaptations to the drought climate and other extreme environments have been proven in the cultivated species of *E. tef* (Assefa et al., 2015), and indeed, the slime cells around seeds were a possible explanation for *Eragrostis* species growing in arid habitats (Kreitschitz et al., 2009).

In summary, conservatism and adaptation seem paradoxical on the surface. Our results strongly support the view that adaptation and conservatism of genome sizes, which mainly resulted from a large number of polyploidization events, play important roles in the evolution in the genus *Eragrostis*. To cope with the environmental changes, as an evolutionary consequence of polyploidization, genome sizes have varied largely and evolved adaptively on one hand. On the other hand, phylogenetic conservatism of the genus may have provided a certain degree of gene flow, accelerated the drought resistance genes to be transferred across species, and finally led to a worldwide distribution of *Eragrostis* species in the arid area. Indeed, the mechanisms of genome size variation and evolution in *Eragrostis* remain largely unsolved, awaiting for the availability of several high-quality reference genomes. We hope that this study will help to understand the adaptive potentials of *Eragrostis* in the exploitation of arid areas, thereby promoting the crop and livestock in the future.

## Data availability statement

The datasets presented in this study can be found in online repositories. The names of the repository/repositories and accession number(s) can be found in the article/**Supplementary Material**.

## Author contributions

LG, G-RH, and YT planed and designed the research. G-RH, YT, and X-GZ conducted the experiments. G-RH analyzed the data, LG and G-RH wrote and revised the manuscript. All authors contributed to the article and approved the submitted version.
