# Supplementary material for: Genome size variation and polyploidy prevalence in the genus Eragrostis are associated with the global dispersal in arid area
Source: Front Plant Sci. 2023 Mar 13;14:1066925. doi: 10.3389/fpls.2023.1066925 (PMC10040770; doi:10.3389/fpls.2023.1066925)
Supplement: Supplementary file 2 [file DataSheet_1.doc]

**Supplementary materials**

**Table S1** DNA contents of 18 *Eragrostis tef* (*E. tef*) cultivars and 2 individuals of *Eragrostis cylindriflora*.

| **Species** | **2C (pg)** | **SD (pg)** | **1C (Mbp)** |
| --- | --- | --- | --- |
|
| *Eragrostis cylindriflora* 2 | 1.64 | 0.04 | 803.22 |
| *Eragrostis cylindriflora* 3 | 1.92 | 0.06 | 939.52 |
| *E. tef* ‘Ada’ | 1.23 | 0.12 | 601.88 |
| *E. tef* ‘Addisie’ | 1.28 | 0.08 | 624.16 |
| *E. tef* ‘Alba’ | 1.31 | 0.16 | 640.73 |
| *E. tef* ‘Balami’ | 1.29 | 0.16 | 630.87 |
| *E. tef* ‘Beten’ | 1.31 | 0.15 | 641.85 |
| *E. tef* ‘Dabbi’ | 1.29 | 0.13 | 629.31 |
| *E. tef* ‘Enatite’ | 1.28 | 0.05 | 626.96 |
| *E. tef* ‘Gealamie’ | 1.22 | 0.01 | 596.71 |
| *E. tef* ‘Gommadie’ | 1.34 | 0.13 | 655.36 |
| *E. tef* ‘Gommandie’ | 1.31 | 0.13 | 640.06 |
| *E. tef* ‘Karadebi’ | 1.31 | 0.07 | 642.53 |
| *E. tef* ‘Kaymuri’ | 1.21 | 0.07 | 591.05 |
| *E. tef* ‘Magna’ | 1.25 | 0.05 | 612.02 |
| *E. tef* ‘Manjna’ | 1.21 | 0.04 | 590.91 |
| *E. tef* ‘Manyi’ | 1.27 | 0.12 | 622.2 |
| *E. tef* ‘RedDabbi’ | 1.28 | 0.06 | 627.87 |
| *E. tef* ‘Tsedey’ | 1.29 | 0.06 | 628.86 |
| *E. tef* ‘TulluNasy’ | 1.29 | 0.1 | 628.76 |

**Table S2** Primer pairs used for PCR experiments and sequencing in this study.

| **Region** | **Primers** | **5’ sequence 3’** | **Annealing temperature** | **Reference^a^** |
| --- | --- | --- | --- | --- |
| *rbcL* | 1f | ATGTCACCACAAACAGAAAC | 45 | Kress and Erickson, 2007 |
|  | 724r | TCGCATGTACCTGCAGTAGC |  |  |
| *matK* | M3 | GCAACAATACTTCCTATATCCGCTT | 40-45 | CBOL (http://barcoding.si.edu) |
|  | M4 | GAACTCTTCTAATAATCCCGAACCT |  |  |
| *trnL-trnF* | f | ATTTGAACTGGTGACACACGAG | 45 | Taberlet et al., 1991 |
|  | c | CGAAATCGGTAGACGCTACG |  |  |
| ITS^b^ | ny47 | AACAAGGTTTCCGTAGGTGA | 45 | Fu et al., 2005 |
|  | Ny207 | CAGTGCCTCGTGGTGCGACA |  |  |

^a^Primers from references as below:

Fu, C., Kong, H., Qiu, Y., Cameron, K. M. (2005). Molecular phylogeny of the East Asian–North American disjunct *Smilax* sect. *Nemexia* (Smilacaceae). *Int. J. Plant Sci.* 166, 301-309. doi: 10.1086/427200

Kress, W. J., Erickson, D. L. (2007). A two-locus global DNA barcode for land plants: the coding *rbcL* gene complements the non-coding *trnH*-*psbA* spacer region. *PLoS One* 2, e508. doi: 10.1371/journal.pone.0000508

Taberlet, P., Gielly, L., Pautou, G., Bouvet, J. (1991). Universal primers for amplification of three non-coding regions of chloroplast DNA. *Plant Mol. Bio*l. 17, 1105-1109. doi: 10.1007/BF00037152

^b^ITS1, 5.8S, and ITS2

**Table S3** Information of NCBI accession numbers for the four outgroup species.

| **Outgroup species** | ***rbcL*** | ***matK*** | ***trnL-trnF*** | **ITS** |
| --- | --- | --- | --- | --- |
| *Cottea pappophoroides* | JN681660 | AF312359 | EF156682 | EF153033 |
| *Enneapogon desvauxii* | MF114365 | JN681629 | EF156692 | MF029719 |
| *Tetrachne dregei* | JN681706 | AF312363 | GU990366 | GU359218 |
| *Uniola paniculata* | AY632373 | AF144607 | GU990369 | GU359192 |

**Table S5** The occupied continents and life histories of *Eragrostis* species.

| **Species** | **Occupied continents** | **Life**  **history^b^** | **Species** | **Occupied**  **continents** | **Life**  **history^b^** |
| --- | --- | --- | --- | --- | --- |
| *Eragrostis acutiflora* | 2 | P | *Eragrostis nigra* | 1 | A |
| *Eragrostis acutiglumis* | 1 | P | *Eragrostis nindensis* | 1 | P |
| *Eragrostis aethiopica* | 1 | A | *Eragrostis nutans* | 1 | P |
| *Eragrostis bahiensis* | 2 | P | *Eragrostis obtusa* | 1 | P |
| *Eragrostis barbinodis* | 1 | B | *Eragrostis papposa* | 4 | B |
| *Eragrostis bicolor* | 1 | P | *Eragrostis patens* | 1 | A |
| *Eragrostis cilianensis* | 6 | A | *Eragrostis patentipilosa* | 1 | B |
| *Eragrostis cylindriflora* | 3 | B | *Eragrostis patentissima* | 1 | P |
| *Eragrostis dielsii* | 1 | B | *Eragrostis pilosa* | 6 | A |
| *Eragrostis echinochloidea* | 2 | P | *Eragrostis plana* | 3 | P |
| *Eragrostis eriopoda* | 1 | P | *Eragrostis polytricha* | 2 | P |
| *Eragrostis ferruginea* | 1 | P | *Eragrostis porosa* | 1 | A |
| *Eragrostis gummiflua* | 1 | P | *Eragrostis racemosa* | 1 | P |
| *Eragrostis heteromera* | 1 | P | *Eragrostis rotifer* | 1 | P |
| *Eragrostis humidicola* | 1 | P | *Eragrostis rufescens* | 3 | A |
| *Eragrostis intermedia* | 2 | P | *Eragrostis sarmentosa* | 1 | P |
| *Eragrostis japonica* | 6 | B | *Eragrostis spectabilis* | 3 | P |
| *Eragrostis lappula* | 1 | P | *Eragrostis superba* | 4 | P |
| *Eragrostis lehmanniana* | 3 | P | *Eragrostis tef* | 6 | A |
| *Eragrostis leptocarpa* | 1 | B | *Eragrostis tenella* | 6 | A |
| *Eragrostis lugens* | 4 | P | *Eragrostis tenuifolia* | 5 | P |
| *Eragrostis virescens* | 6 | A | *Eragrostis tremula* | 2 | B |
| *Eragrostis minor* | 6 | A | *Eragrostis unioloides* | 5 | B |
| *Eragrostis neesii* | 1 | B |  |  |  |

^a^ The 1-6 represented the number of continents occupied by each species;

^b^ Life history: P, perennial; A, annual; B, biennial.

**Table S6** Likelihood ratio tests (LRTs) between the evolutionary models ^a, b^.

| **Traits** | **LRTs  (BM, OU)** | **LRTs  (Delta, BM)** | **LRTs  (Lambda, No-signal)** |
| --- | --- | --- | --- |
| Genome size | 0.334 | < 0.001*** | 0.007** |
| Annual Mean Temperature | 0.307 | < 0.001*** | 1 |
| Mean Diurnal Range | 0.307 | < 0.001*** | 1 |
| Isothermality | 0.307 | < 0.001*** | 1 |
| Temperature Seasonality | 0.307 | 0.006** | 1 |
| Max Temperature of Warmest Month | 0.307 | < 0.001*** | 1 |
| Min Temperature of Coldest Month | 0.307 | 0.036* | 1 |
| Temperature Annual Range | 0.307 | 0.099 | 1 |
| Mean Temperature of Wettest Quarter | 0.308 | 0.001** | 1 |
| Mean Temperature of Driest Quarter | 0.307 | 0.001** | 1 |
| Mean Temperature of Warmest Quarter | 0.308 | < 0.001*** | 0.616 |
| Mean Temperature of Coldest Quarter | 0.307 | < 0.001*** | 1 |
| Annual Precipitation | 0.307 | < 0.001*** | 1 |
| Precipitation of Wettest Month | 0.307 | 0.005** | 1 |
| Precipitation of Driest Month | 0.308 | < 0.001*** | 1 |
| Precipitation Seasonality | 0.307 | < 0.001*** | 1 |
| Precipitation of Wettest Quarter | 0.307 | 0.003** | 1 |
| Precipitation of Driest Quarter | 0.307 | < 0.001*** | 1 |
| Precipitation of Warmest Quarter | 0.307 | < 0.001*** | 1 |
| Precipitation of Coldest Quarter | 0.308 | 0.002** | 1 |
| Latitude | 0.308 | 0.076 | 1 |
| Longitude | 0.307 | < 0.001*** | 0.472 |
| Elevation | 0.320 | < 0.001*** | 0.798 |
| SNBT | 0.308 | 0.003** | 1 |
| SNBP | 0.307 | 0.095 | 1 |
| WLNBT | 0.307 | 0.110 | 1 |
| WLNBP | 0.307 | 0.008** | 1 |

^a^ When the *p* values of LRTs showed no significance, simplest models were selected.

^b^ LRTs, likelihood ratio tests. BM, Brownian motion; OU, Ornstein-Uhlenbeck; No-signal, λ=0.

**Table S7** BayesTraits used to test the observed and expected values of evolutionary parameters in different models of trait evolution.

| **Trait** | **Value** | **Lh** | **Log marginal likelihood** | **Log BF** | **Trait** | **Value** | **Lh** | **Log marginal likelihood** | **Log BF** |
| --- | --- | --- | --- | --- | --- | --- | --- | --- | --- |
| **Genome size** | | | | | **Annual precipitation** | | | | |
| Lambda λ |  |  |  |  | Lambda λ |  |  |  |  |
| **λ Estimated** | **0.75** | **-47.66** | **-53.03** |  | **λ Estimated** | **0.00** | **-354.95** | **-417.49** |  |
| λ Forced = 1 |  | -70.16 | -74.27 | >10 | λ Forced = 1 |  | -512.20 | -512.20 | >10 |
| λ Forced = 0 |  | -51.26 | -57.78 | 5-10 | **λ Forced = 0** |  | **-354.95** | **-417.55** | **<2** |
| Delta δ |  |  |  |  | Delta δ |  |  |  |  |
| **δ Estimated** | **3.00** | **-62.76** | **-67.95** |  | **δ Estimated** | **3.00** | **-506.60** | **-506.61** |  |
| δ Forced =1 |  | -70.16 | -74.32 | >10 | δ Forced =1 |  | -512.20 | -512.20 | >10 |
| δ Forced =0 |  | -88.96 | -88.96 | >10 | δ Forced =0 |  | -530.01 | -530.01 | >10 |
| Kappa κ |  |  |  |  | Kappa κ |  |  |  |  |
| **κ Estimated** | **0.43** | **-46.08** | **-51.04** |  | **κ Estimated** | **0.00** | **-360.38** | **-361.86** |  |
| κ Forced =1 |  | -70.16 | -74.36 | >10 | κ Forced =1 |  | -512.20 | -512.20 | >10 |
| κ Forced =0 |  | -48.95 | -54.45 | 5-10 | **κ Forced =0** |  | **-360.38** | **-361.86** | **<2** |
| **Latitude** | | | | | **Mean Diurnal Range** | | | | |
| Lambda λ |  |  |  |  | Lambda λ |  |  |  |  |
| **λ Estimated** | **0.00** | **-222.19** | **-225.19** |  | **λ Estimated** | **0.00** | **-108.71** | **-114.04** |  |
| λ Forced = 1 |  | -328.29 | -328.31 | >10 | λ Forced = 1 |  | -265.94 | -266.18 | >10 |
| **λ Forced = 0** |  | **-222.19** | **-225.19** | **<2** | **λ Forced = 0** |  | **-108.71** | **-114.18** | **<2** |
| Delta δ |  |  |  |  | Delta δ |  |  |  |  |
| **δ Estimated** | **2.89** | **-326.72** | **-326.83** |  | **δ Estimated** | **3.00** | **-260.34** | **-261.34** |  |
| δ Forced =1 |  | -328.29 | -328.31 | >2 | δ Forced =1 |  | -265.94 | -266.18 | 5-10 |
| δ Forced =0 |  | -343.58 | -343.58 | >10 | δ Forced =0 |  | -283.75 | -283.75 | >10 |
| Kappa κ |  |  |  |  | Kappa κ |  |  |  |  |
| **κ Estimated** | **0.00** | **-231.48** | **-233.01** |  | **κ Estimated** | **0.00** | **-119.04** | **-122.99** |  |
| κ Forced =1 |  | -328.29 | -328.31 | >10 | κ Forced =1 |  | -265.94 | -266.18 | >10 |
| **κ Forced =0** |  | **-231.48** | **-233.04** | **<2** | **κ Forced =0** |  | **-119.04** | **-123.07** | **<2** |
| **Longitude** | | | | | **SNBT** | | | | |
| Lambda λ |  |  |  |  | Lambda λ |  |  |  |  |
| **λ Estimated** | **0.18** | **-255.64** | **-257.31** |  | **λ Estimated** | **0.00** | **-187.20** | **-190.87** |  |
| λ Forced = 1 |  | -386.34 | -386.34 | >10 | λ Forced = 1 |  | -285.68 | -285.85 | >10 |
| **λ Forced = 0** |  | **-255.90** | **-258.20** | **<2** | **λ Forced = 0** |  | **-187.20** | **-191.01** | **<2** |
| Delta δ |  |  |  |  | Delta δ |  |  |  |  |
| **δ Estimated** | **3.00** | **-377.59** | **-377.61** |  | **δ Estimated** | **3.00** | **-281.40** | **-282.12** |  |
| δ Forced =1 |  | -386.34 | -386.34 | >10 | δ Forced =1 |  | -285.68 | -285.85 | 5-10 |
| δ Forced =0 |  | -405.69 | -405.69 | >10 | δ Forced =0 |  | -302.73 | -302.73 | >10 |
| Kappa κ |  |  |  |  | Kappa κ |  |  |  |  |
| **κ Estimated** | **0.00** | **-265.53** | **-266.90** |  | **κ Estimated** | **0.00** | **-192.02** | **-194.38** |  |
| κ Forced =1 |  | -386.34 | -386.34 | >10 | κ Forced =1 |  | -285.68 | -285.85 | >10 |
| **κ Forced =0** |  | **-265.53** | **-266.91** | **<2** | **κ Forced =0** |  | **-192.02** | **-194.42** | **<2** |
| **BayesTraits model** |  |  |  |  |  |  |  |  |  |
| Model B | **-** | -70.09  -70.15 | -74.62 |  |  |  |  |  |  |
| **Model A** | **-** | **-70.15** | **-74.26** | **<2** |  |  |  |  |  |

The selected models are indicated in bold; Log BF, Log Bayes Factors; Lh: log likelihood;

λ = 1, pure Brownian motion; λ = 0, no phylogenetic signal; κ = 1, gradual evolution; κ = 0, punctuated evolution; δ = 1, gradual evolution.

**Table S8** Evolutionary models selection for the environmental factors.

| **Model** | **Parameter** | **AICc** | **Lh** | **Model** | **Parameter** | **AICc** | **Lh** | ***k*** |
| --- | --- | --- | --- | --- | --- | --- | --- | --- |
| **Annual Mean Temperature** | | | | **Precipitation of Driest Month** | | | | |
| BM |  | 531.56 | -263.64 | BM |  | 642.81 | -319.27 | 2 |
| Lambda | λ = 0.00 | 270.27 | -131.85 | Lambda | λ = 0.00 | 405.95 | -199.69 | 3 |
| Delta | δ = 2.99 | 521.45 | -257.45 | Delta | δ = 2.99 | 632.69 | -313.07 | 3 |
| Kappa | κ = 0.00 | 290.12 | -141.78 | Kappa | κ = 0.00 | 417.95 | -205.70 | 3 |
| **No-signal** | **λ forced = 0.00** | **267.98** | **-131.85** | **No-signal** | **λ forced = 0.00** | **403.66** | **-199.69** | **2** |
| OU | α = 2.72 | 532.80 | -263.12 | OU | α = 2.72 | 644.06 | -318.75 | 3 |
| **Isothermality** | | | | **Precipitation Seasonality** | | | | |
| BM |  | 612.02 | -303.87 | BM |  | 717.88 | -356.80 | 2 |
| Lambda | λ = 0.00 | 361.31 | -177.38 | Lambda | λ = 0.00 | 440.43 | -216.94 | 3 |
| Delta | δ = 2.99 | 587.30 | -290.37 | Delta | δ = 2.99 | 692.90 | -343.17 | 3 |
| Kappa | κ = 0.00 | 384.00 | -188.72 | Kappa | κ = 0.00 | 461.07 | -227.25 | 3 |
| **No-signal** | **λ forced = 0.00** | **359.03** | **-177.38** | **No-signal** | **λ forced = 0.00** | **438.15** | **-216.94** | **2** |
| OU | α = 2.72 | 613.27 | -303.35 | OU | α = 2.72 | 719.12 | -356.28 | 3 |
| **Temperature Seasonality** | | | | **Precipitation of Wettest Quarter** | | | | |
| BM |  | 870.43 | -433.08 | BM |  | 925.80 | -474.26 | 2 |
| Lambda | λ = 0.00 | 623.77 | -308.61 | Lambda | λ = 0.00 | 637.94 | -315.69 | 3 |
| Delta | δ = 2.99 | 865.11 | -429.28 | Delta | δ = 2.99 | 946.13 | -469.78 | 3 |
| Kappa | κ = 0.03 | 643.94 | -318.69 | Kappa | κ = 0.00 | 655.20 | -324.32 | 3 |
| **No-signal** | **λ forced = 0.00** | **621.49** | **-308.61** | **No-signal** | **λ forced = 0.00** | **635.65** | **-315.69** | **2** |
| OU | α = 2.72 | 871.67 | -432.56 | OU | α = 2.72 | 954.04 | -473.74 | 3 |
| **Max Temperature of Warmest Month** | | | | **Precipitation of Driest Quarter** | | | | |
| BM |  | 509.14 | -252.43 | BM |  | 770.92 | -383.33 | 2 |
| Lambda | λ = 0.00 | 253.12 | -123.28 | Lambda | λ = 0.00 | 522.08 | -257.76 | 3 |
| Delta | δ = 2.99 | 484.11 | -238.77 | Delta | δ = 2.99 | 760.80 | -377.12 | 3 |
| Kappa | κ = 0.00 | 266.95 | -130.20 | Kappa | κ = 0.00 | 533.08 | -263.26 | 3 |
| **No-signal** | **λ forced = 0.00** | **250.83** | **-123.28** | **No-signal** | **λ forced = 0.00** | **519.79** | **-257.76** | **2** |
| OU | α = 2.72 | 510.38 | -251.91 | OU | α = 2.72 | 772.17 | -382.80 | 3 |
| **Min Temperature of Coldest Month** | | | | **Precipitation of Warmest Quarter** | | | | |
| BM |  | 576.00 | -285.87 | BM |  | 941.26 | -468.50 | 2 |
| Lambda | λ = 0.00 | 299.82 | -146.63 | Lambda | λ = 0.00 | 611.01 | -302.23 | 3 |
| Delta | δ = 2.99 | 573.91 | -283.68 | Delta | δ = 2.99 | 925.88 | -459.66 | 3 |
| Kappa | κ = 0.00 | 316.11 | -154.78 | Kappa | κ = 0.00 | 634.16 | -313.80 | 3 |
| **No-signal** | **λ forced = 0.00** | **297.53** | **-146.63** | **No-signal** | **λ forced = 0.00** | **608.73** | **-302.23** | **2** |
| OU | α = 2.72 | 577.25 | -285.34 | OU | α = 2.72 | 942.51 | -467.97 | 3 |
| **Temperature Annual Range** | | | | **Precipitation of Coldest Quarter** | | | | |
| BM |  | 574.80 | -285.27 | BM |  | 811.54 | -403.63 | 2 |
| Lambda | λ = 0.00 | 293.30 | -143.37 | Lambda | λ = 0.00 | 576.44 | -284.94 | 3 |
| Delta | δ = 2.74 | 574.39 | -283.91 | Delta | δ = 2.99 | 804.06 | -398.75 | 3 |
| Kappa | κ = 0.00 | 309.09 | -151.26 | Kappa | κ = 0.00 | 586.32 | -289.88 | 3 |
| **No-signal** | **λ forced = 0.00** | **291.01** | **-143.37** | **No-signal** | **λ forced = 0.00** | **574.16** | **-284.9** | **2** |
| OU | α = 2.72 | 576.05 | -284.74 | OU | α = 2.72 | 812.79 | -403.11 | 3 |
| **Mean Temperature of Wettest Quarter** | | | | **Longitude** | | | | |
| BM |  | 455.59 | -225.66 | BM |  | 776.95 | -386.34 | 2 |
| Lambda | λ = 0.00 | 258.33 | -125.88 | Lambda | λ = 0.18 | 517.83 | -255.64 | 3 |
| Delta | δ = 2.99 | 447.22 | -220.33 | Delta | δ = 2.99 | 761.73 | -377.58 | 3 |
| Kappa | κ = 0.00 | 274.83 | -134.14 | Kappa | κ = 0.00 | 537.62 | -265.53 | 3 |
| **No-signal** | **λ forced = 0.00** | **256.04** | **-125.88** | **No-signal** | **λ forced = 0.00** | **516.06** | **-255.89** | **2** |
| OU | α = 2.72 | 456.84 | -225.14 | OU | α = 2.72 | 778.19 | -385.82 | 3 |
| **Mean Temperature of Driest Quarter** | | | | **Elevation** | | | | |
| BM |  | 572.46 | -284.09 | BM |  | 802.66 | -399.19 | 2 |
| Lambda | λ = 0.00 | 299.50 | -146.47 | Lambda | λ = 0.09 | 714.83 | -354.13 | 3 |
| Delta | δ = 2.99 | 564.01 | -278.73 | Delta | δ = 2.99 | 788.13 | -390.78 | 3 |
| Kappa | κ = 0.00 | 322.20 | -157.82 | Kappa | κ = 0.00 | 724.96 | -359.19 | 3 |
| **No-signal** | **λ forced = 0.00** | **297.22** | **-146.47** | **No-signal** | **λ forced = 0.00** | **712.61** | **-354.16** | **2** |
| OU | α = 2.72 | 573.70 | -283.57 | OU | α = 2.72 | 803.96 | -398.70 | 3 |
| **Mean Temperature of Warmest Quarter** | | | | **SNBP** | | | | |
| BM |  | 470.40 | -233.06 | BM |  | 1058.05 | -526.89 | 2 |
| Lambda | λ = 0.12 | 242.17 | -117.81 | Lambda | λ = 0.00 | 755.60 | -374.52 | 3 |
| Delta | δ = 2.99 | 459.34 | -226.40 | Delta | δ = 2.77 | 1057.55 | -525.50 | 3 |
| Kappa | κ = 0.00 | 255.11 | -124.28 | Kappa | κ = 0.00 | 773.77 | -383.61 | 3 |
| **No-signal** | **λ forced = 0.00** | **240.14** | **-117.93** | **No-signal** | **λ forced = 0.00** | **753.31** | **-374.52** | **2** |
| OU | α = 2.72 | 471.64 | -232.54 | OU | α = 2.72 | 1059.29 | -526.37 | 3 |
| **Mean Temperature of Coldest Quarter** | | | | **WLNBT** | | | | |
| BM |  | 568.58 | -282.16 | BM |  | 565.52 | -280.63 | 2 |
| Lambda | λ = 0.00 | 300.74 | -147.09 | Lambda | λ = 0.00 | 290.71 | -142.08 | 3 |
| Delta | δ = 2.99 | 559.16 | -276.30 | Delta | δ = 2.69 | 565.25 | -279.35 | 3 |
| Kappa | κ = 0.00 | 322.01 | -157.72 | Kappa | κ = 0.00 | 305.35 | -149.40 | 3 |
| **No-signal** | **λ forced = 0.00** | **298.46** | **-147.09** | **No-signal** | **λ forced = 0.00** | **288.42** | **-142.08** | **2** |
| OU | α = 2.72 | 569.83 | -281.63 | OU | α = 2.72 | 566.77 | -280.10 | 3 |
| **Precipitation of Wettest Month** | | | | **WLNBP** | | | | |
| BM |  | 853.93 | -424.83 | BM |  | 927.65 | -461.69 | 2 |
| Lambda | λ = 0.00 | 543.46 | -268.45 | Lambda | λ = 0.00 | 619.27 | -306.36 | 3 |
| Delta | δ = 2.99 | 848.22 | -420.83 | Delta | δ = 2.99 | 922.84 | -458.14 | 3 |
| Kappa | κ = 0.00 | 560.11 | -276.78 | Kappa | κ = 0.00 | 628.56 | -316.00 | 3 |
| **No-signal** | **λ forced = 0.00** | **541.18** | **-268.45** | **No-signal** | **λ forced = 0.00** | **616.99** | **-306.36** | **2** |
| OU | α = 2.72 | 855.18 | -424.31 | OU | α = 2.72 | 928.90 | -461.17 | 3 |

The selected models are indicated in bold; Lh: log likelihood; AICc, corrected AIC value; *k*, free parameters

**Table S9** Correlations between genome sizes and environmental factors estimated by three types of regression models.

| **Model** | ***R^2^*** | ***b*** | ***P*** | **AIC** | **Log likelihood** | **LRTs** |
| --- | --- | --- | --- | --- | --- | --- |
| Annual Mean Temperature-Genome size |  |  |  |  |  |  |
| **PGLS** | **0.021** | **-0.206** | **0.333** | **47.25** | **-21.63** |  |
| OLS | ~0.000 | -0.018 | 0.945 | 53.45 | -24.73 | <0.001^***^ |
| PIC | 0.002 | 0.007 | 0.793 | 80.72 | -38.36 | <0.001^***^ |
| Isothermality-Genome size |  |  |  |  |  |  |
| **PGLS** | **0.035** | **-0.288** | **0.207** | **46.54** | **-21.27** |  |
| OLS | 0.009 | -0.181 | 0.530 | 53.04 | -24.52 | <0.001^***^ |
| PIC | 0.329 | 0.096 | <0.001^***^ | 62.05 | -29.02 | <0.001^***^ |
| Temperature Seasonality-Genome size |  |  |  |  |  |  |
| **PGLS** | **0.039** | **0.133** | **0.182** | **46.29** | **-21.15** |  |
| OLS | 0.020 | 0.113 | 0.337 | 52.48 | -24.24 | <0.001^***^ |
| PIC | 0.001 | 0.003 | 0.811 | 80.74 | -38.37 | <0.001^***^ |
| Max Temperature of Warmest Month -genome size |  |  |  |  |  |  |
| **PGLS** | **0.002** | **-0.139** | **0.769** | **48.08** | **-22.04** |  |
| OLS | 0.007 | 0.298 | 0.589 | 53.15 | -24.57 | <0.001^***^ |
| PIC | 0.377 | 0.228 | <0.001^***^ | 58.53 | -27.26 | <0.001^***^ |
| Min Temperature of Coldest Month-Genome size |  |  |  |  |  |  |
| **PGLS** | **0.015** | **-0.036** | **0.439** | **43.28** | **-19.64** |  |
| OLS | 0.004 | -0.027 | 0.676 | 50.05 | -23.03 | <0.001^***^ |
| PIC | 0.121 | -0.009 | 0.022^*^ | 68.17 | -32.08 | <0.001^***^ |
| Temperature Annual Range-Genome size |  |  |  |  |  |  |
| **PGLS** | **0.058** | **0.367** | **0.102** | **45.37** | **-20.68** |  |
| OLS | 0.040 | 0.36 | 0.177 | 51.53 | -23.76 | <0.001^***^ |
| PIC | 0.297 | 0.065 | <0.001^***^ | 64.22 | -30.11 | <0.001^***^ |
| Mean Temperature of Wettest Quarter-Genome size |  |  |  |  |  |  |
| **PGLS** | **0.006** | **0.166** | **0.592** | **47.87** | **-21.94** |  |
| OLS | 0.024 | 0.375 | 0.302 | 52.33 | -24.16 | <0.001^***^ |
| PIC | 0.001 | 0.015 | 0.829 | 80.75 | -38.37 | <0.001^***^ |
| Mean Temperature of Warmest Quarter-Genome size |  |  |  |  |  |  |
| **PGLS** | **0.002** | **-0.113** | **0.788** | **48.09** | **-22.05** |  |
| OLS | 0.004 | 0.208 | 0.663 | 53.25 | -24.63 | <0.001^***^ |
| PIC | 0.008 | 0.038 | 0.555 | 80.43 | -38.21 | <0.001^***^ |
| Mean Temperature of Coldest Quarter-Genome size |  |  |  |  |  |  |
| **PGLS** | **0.063** | **-0.130** | **0.089** | **45.27** | **-20.64** |  |
| OLS | 0.008 | -0.051 | 0.552 | 53.08 | -24.54 | <0.001^***^ |
| PIC | ~0.000 | -0.001 | 0.938 | 80.79 | -38.39 | <0.001^***^ |
| Precipitation of Wettest Month-Genome size |  |  |  |  |  |  |
| **PGLS** | **0.004** | **-0.044** | **0.672** | **47.97** | **-21.99** |  |
| OLS | ~0.000 | -0.020 | 0.865 | 53.42 | -24.71 | <0.001^***^ |
| PIC | 0.445 | -0.031 | <0.001^***^ | 53.11 | -24.55 | <0.001^***^ |
| Precipitation of Driest Month-Genome size |  |  |  |  |  |  |
| **PGLS** | **~0.000** | **-0.004** | **0.837** | **48.12** | **-22.06** |  |
| OLS | ~0.000 | -0.004 | 0.888 | 53.43 | -24.72 | <0.001^***^ |
| PIC | 0.443 | -0.003 | <0.001^***^ | 53.30 | -24.65 | <0.001^***^ |
| Precipitation Seasonality-Genome size |  |  |  |  |  |  |
| **PGLS** | **~0.000** | **-0.016** | **0.896** | **48.14** | **-22.07** |  |
| OLS | 0.002 | 0.040 | 0.774 | 53.37 | -24.68 | <0.001^***^ |
| PIC | 0.334 | 0.068 | <0.001^***^ | 61.72 | -28.86 | <0.001^***^ |
| Precipitation of Wettest Quarter-Genome size |  |  |  |  |  |  |
| **PGLS** | **0.003** | **-0.040** | **0.697** | **48.00** | **-22.00** |  |
| OLS | ~0.000 | -0.021 | 0.861 | 53.42 | -24.71 | <0.001^***^ |
| PIC | 0.474 | -0.030 | <0.001^***^ | 50.58 | 23.29 | <0.001^***^ |
| Precipitation of Driest Quarter-Genome size |  |  |  |  |  |  |
| **PGLS** | **0.003** | **-0.006** | **0.727** | **48..03** | **-22.02** |  |
| OLS | 0.002 | -0.007 | 0.769 | 53.36 | -24.68 | <0.001^***^ |
| PIC | 0.441 | -0.003 | <0.001^***^ | 53.49 | -24.74 | <0.001^***^ |
| SNBP-Genome size |  |  |  |  |  |  |
| **PGLS** | **0.046** | **-0.124** | **0.148** | **45.95** | **-20.98** |  |
| OLS | 0.026 | -0.121 | 0.275 | 52.20 | -24.10 | <0.001^***^ |
| PIC | 0.110 | -0.013 | 0.023 | 75.33 | -35.66 | <0.001^***^ |
| WLNBP- Genome size |  |  |  |  |  |  |
| **PGLS** | **0.004** | **-0.041** | **0.686** | **47.99** | **-22.00** |  |
| OLS | ~0.000 | -0.017 | 0.875 | 53.43 | -24.71 | <0.001^***^ |
| PIC | 0.397 | -0.031 | <0.001^***^ | 57.02 | -26.51 | <0.001^***^ |
| WLNBT-Genome size |  |  |  |  |  |  |
| **PGLS** | **0.056** | **0.379** | **0.109** | **46.71** | **-21.36** |  |
| OLS | 0.042 | 0.378 | 0.169 | 49.63 | -22.81 | <0.001^***^ |
| PIC | 0.284 | 0.070 | <0.001^***^ | 65.92 | -30.96 | <0.001^***^ |
| Elevation-Genome size |  |  |  |  |  |  |
| **PGLS** | **0.004** | **-0.024** | **0.662** | **48.74** | **-22.44** |  |
| OLS | 0.013 | -0.043 | 0.458 | 52.88 | -24.44 | <0.001^***^ |
| PIC | 0.426 | 0.038 | <0.001^***^ | 53.05 | -24.52 | <0.001^***^ |
| Southern latitude-Genome size |  |  |  |  |  |  |
| **PGLS** | **0.026** | **0.059** | **0.386** | **33.22** | **-14.61** |  |
| **OLS** | **0.026** | **0.059** | **0.386** | **33.22** | **-14.61** | － |
| PIC | 0.014 | 0.058 | 0.532 | 44.48 | -20.24 | <0.001^***^ |
| Western longitude-Genome size |  |  |  |  |  |  |
| **PGLS** | **~0.000** | **0.009** | **0.935** | **13.52** | **-4.76** |  |
| OLS | 0.006 | 0.025 | 0.834 | 15.81 | -5.91 | <0.001^***^ |
| **PIC** | **~0.000** | **0.009** | **0.935** | **13.52** | **-4.76** | － |
| Longitudinal Range-Genome size |  |  |  |  |  |  |
| **PGLS** | **0.014** | **-0.377** | **0.423** | **160.63** | **-78.32** |  |
| **OLS** | **0.014** | **-0.377** | **0.423** | **160.63** | **-78.32** | － |
| PIC | 0.289 | -20.968 | <0.001^***^ | 409.15 | -202.58 | <0.001^***^ |
| Latitudinal range-Genome size |  |  |  |  |  |  |
| **PGLS** | **0.076** | **-0.550** | **0.060** | **114.52** | **-55.26** |  |
| **OLS** | **0.076** | **-0.550** | **0.060** | **114.52** | **-55.26** | － |
| PIC | 0.114 | -6.701 | 0.020^*^ | 355.93 | -175.97 | <0.001^***^ |

LRTs, likelihood ratio tests; AIC, Akaike information criterion.


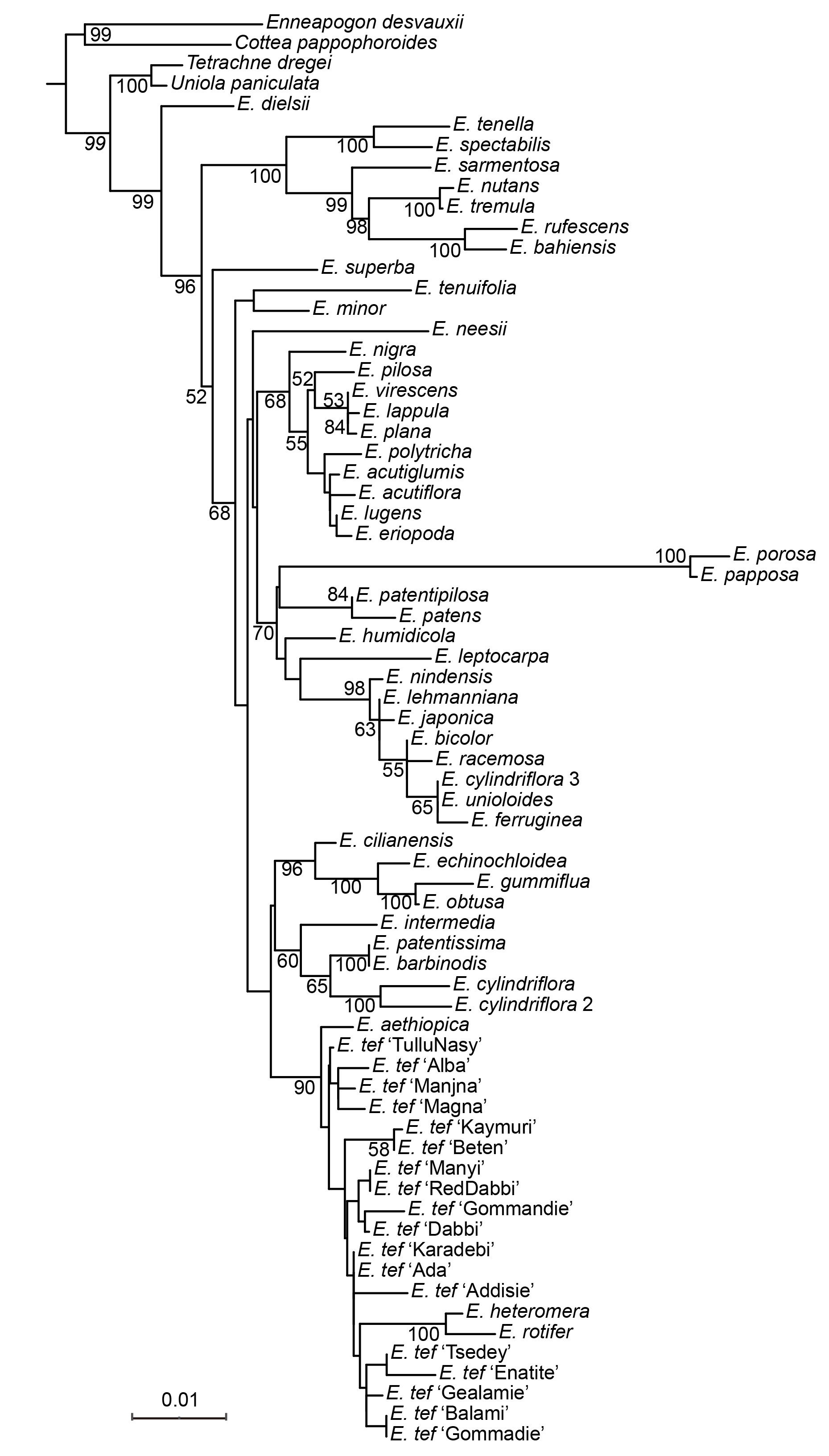


**Figure S1** Phylogram of best maximum likelihood tree from analysis of plastid combined data.


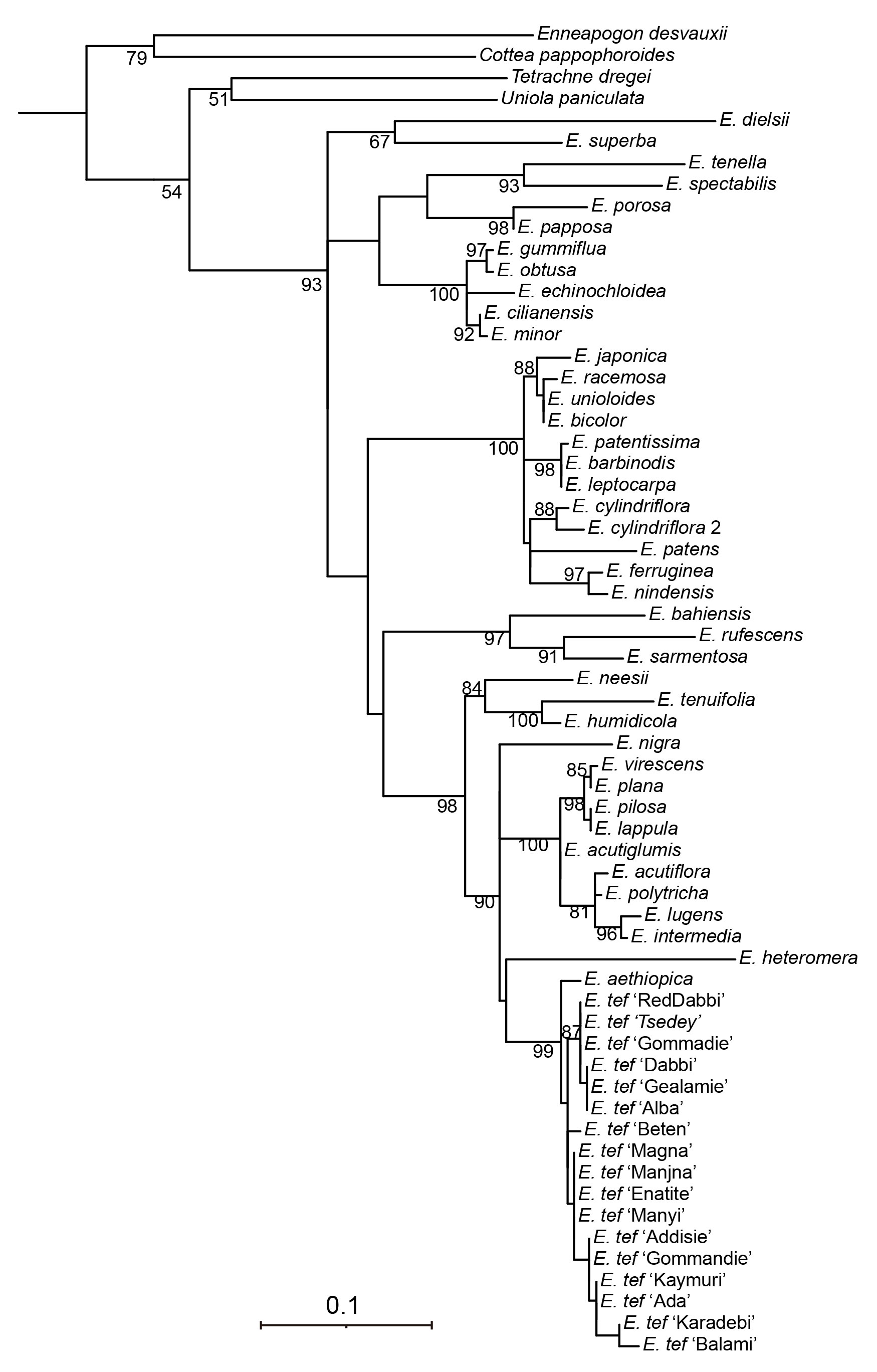


**Figure S2** Phylogram of best maximum likelihood tree from analysis of ITS data.


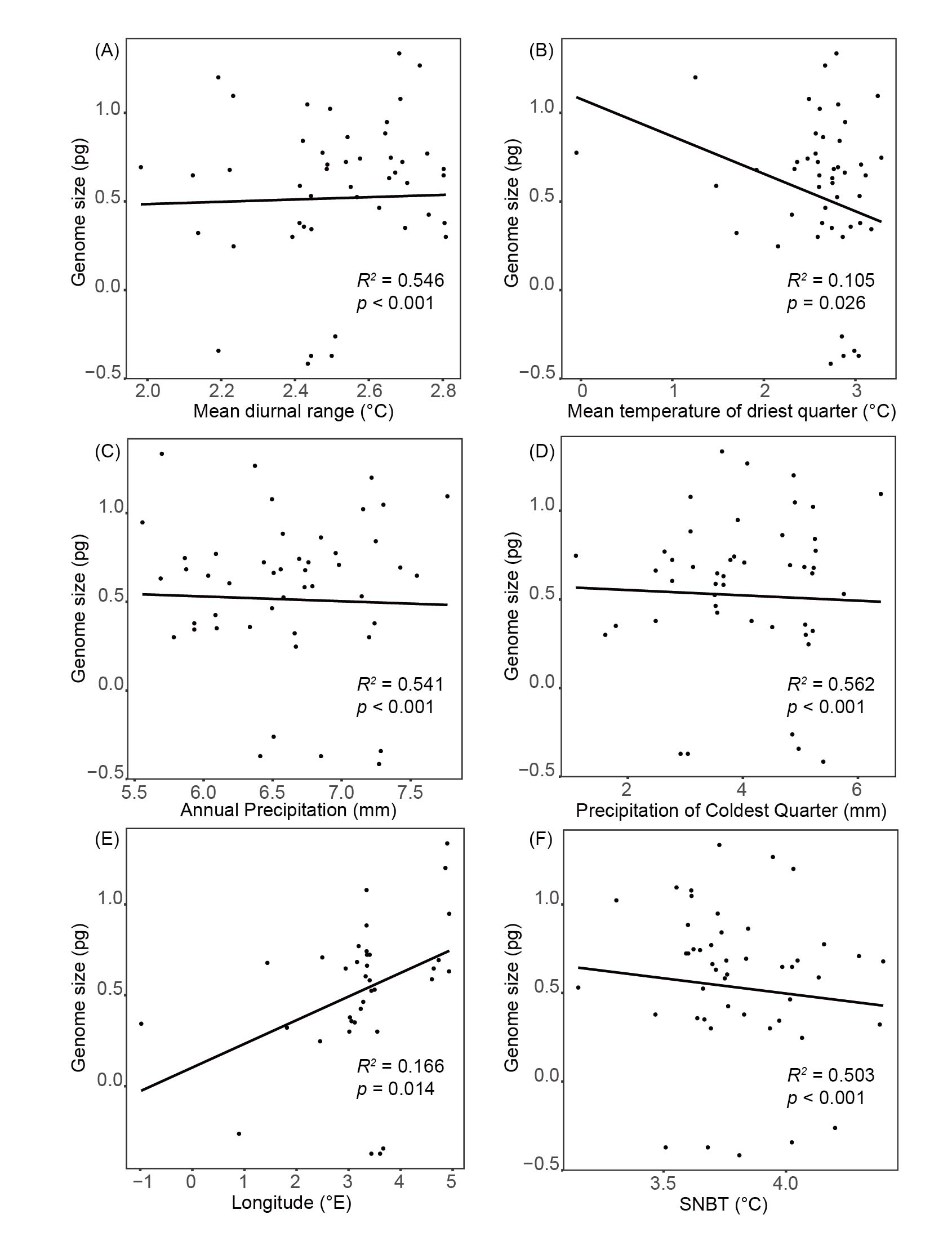


**Figure S3** Scatter plots showed the relationships between genome sizes and (A) mean diurnal range (B) mean temperature of driest quarter (C) annual precipitation (D) precipitation of coldest quarter (E) Eastern longitude (F) SNBT.


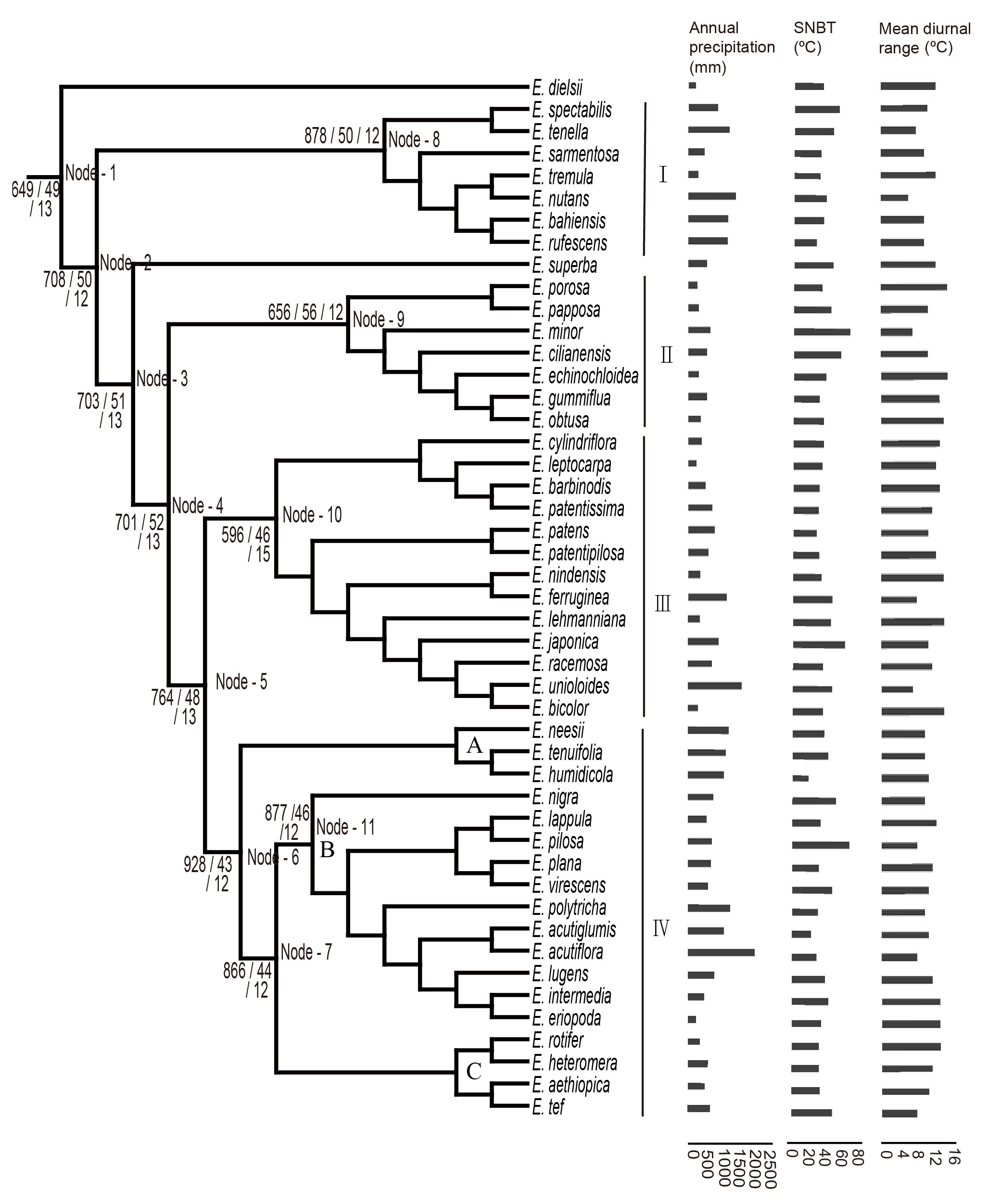


**Figure S4** The phylogram reconstructed by combined ITS and plastid data. The ancestral states of annual precipitation (mm), SNBT (°C) and mean diurnal range (°C) (from left to right). Numbers below major nodes are estimated ancestral values of three bioclimatic niches. *E. tef*: *E. tef* 'RedDabbi'.


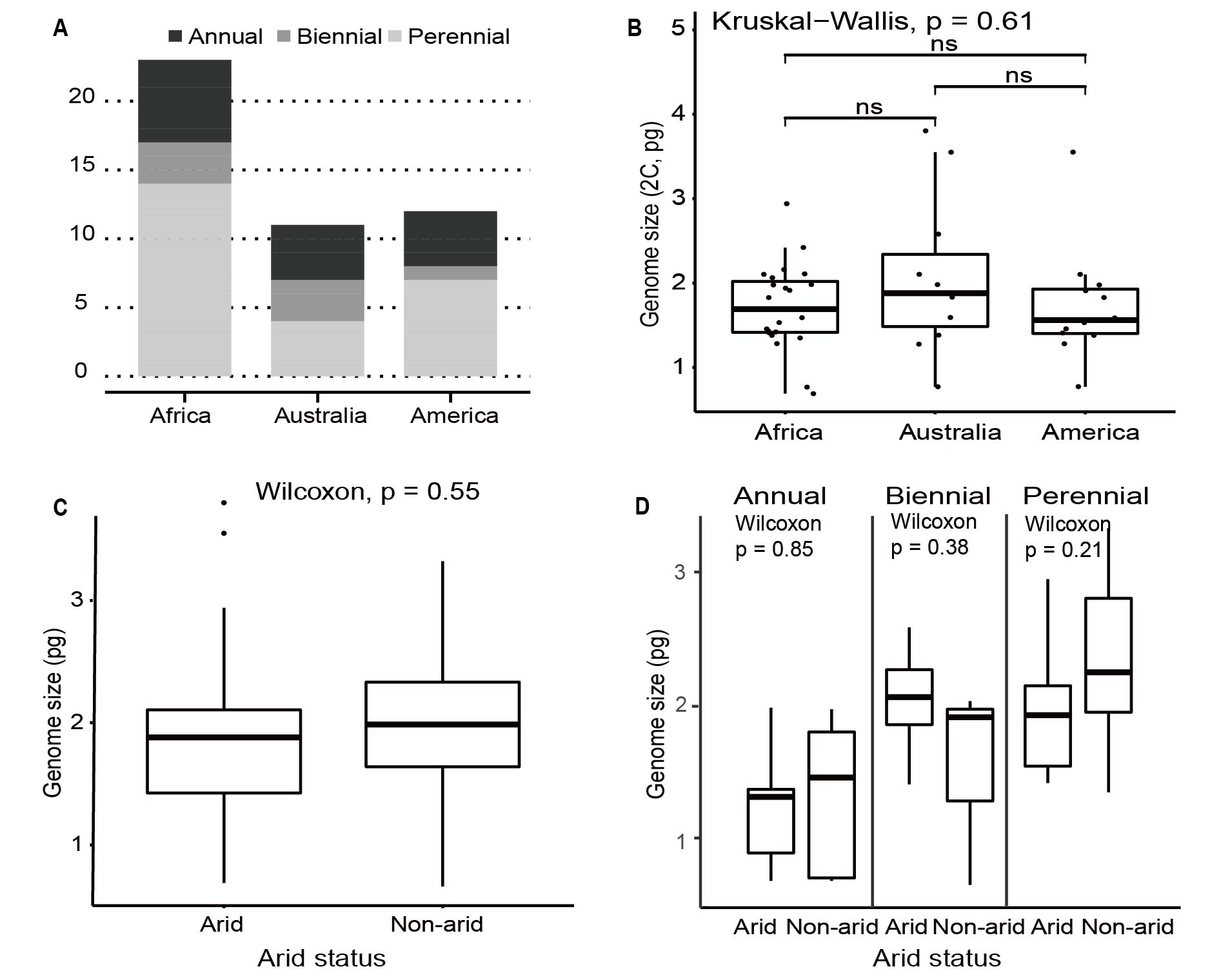


**Figure S5** (A) Histogram of the proportion of different life styles in Africa, America and Australia; (B) genome size variation among Africa, America and Australia; (C) genome size variation among arid and non-arid group; and (D) genome size variation among different life styles.
